# A low-cost, mobile real-time kinematic geolocation service for engineering and research applications

**DOI:** 10.1016/j.ohx.2021.e00203

**Published:** 2021-05-19

**Authors:** André Broekman, Petrus Johannes Gräbe

**Affiliations:** Department of Civil Engineering, Engineering 4.0, University of Pretoria, Pretoria, South Africa

**Keywords:** RTK, GNSS, GPS, NTRIP, PPP, RTK2go, RTK-ASM, UAV, LiDAR, U-blox, Geolocation, Surveying, Point cloud, Photogrammetry

## Abstract

Centimetre accurate geolocation service is beneficial to a wide range of applications, ranging from sports engineering, civil infrastructure, autonomous vehicles, surveying to digitisation of historically significant structures. Previously, these features were confined to prohibitively expensive commercial hardware, requiring technical knowledge and experience to operate. Continued technological advancements have seen the miniaturisation of electronics and antennas, coupled with an increase in the number and performance of global navigation satellite systems (GNSS) by various nations and organisations, providing global signal coverage. This paper demonstrates a low-cost, mobile, real-time kinematic (RTK) geolocation service for engineering and research applications, fabricated from components readily available from commercial suppliers. This solution, consisting of a mobile RTK base station and RTK rover, provides centimetre-accuracy performance up to a distance of 15 km away from the base station. Correction data is transmitted over the internet using free and open software solutions. The small footprint of both the RTK base station and RTK rover, provides versatile applications even in remote locations. The performance of the geolocation service is validated using field experiments, comparing measurements against state-of-the-art photogrammetry, light detection and ranging (LiDAR) and digital level measurement technologies. The authors encourage the adoption of the RTK geolocation solution based on the calibrated results.

## Highlights

• Successful realisation of a low-cost RTK-grade geolocation service.
• Digital twinning of both modern and historically significant assets.
• Mobile acquisition unit incorporating high level customizability.
• Low cost and wide serviceability area for multidisciplinary research.

## Graphical abstract

**Figure:**
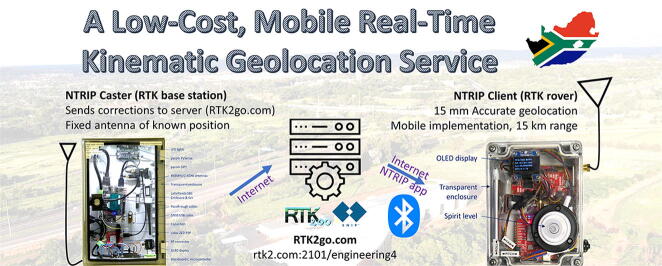

Specifications table

**Table:** 

Hardware name	Real-Time Kinematic Geolocation Service
Subject area	General
Hardware type	Field measurements and sensors
Open Source License	Creative Commons Attribution-ShareAlike
Cost of Hardware	$981.92 USD (RTK base station) $548.92 USD (RTK rover)
Source File Repository	https://doi.org/10.17605/OSF.IO/QH3V7

## 1 Hardware in context

Against the backdrop of the 4th Industrial Revolution (4IR), cost-effective, digital condition monitoring of infrastructure is receiving renewed interest. Rail and road infrastructures constitute key enabling components of smart, optimised and equitable transportation networks of the future [1]. A new generation of millimetre accurate geolocation technologies hold the potential to advance these ambitions for developing more accurate Digital Twins [2] and cost-effective, data-driven maintenance strategies.

A total of six satellite positioning systems, collectively referred to as a Global Navigation Satellite Systems (GNSS), are currently in orbit [3]: GPS (Global Positioning System, United States), GLONASS (GLObal Navigation Satellite System, Russian Federation), Galileo (European Global Navigation Satellite Systems Agency (GSA)), BeiDou (approximately translated to “Northern Dipper”, People’s Republic of China), IRNSS (Indian Regional Navigation Satellite System, India) and QZSS (Quasi-Zenith Satellite System, Japan). The latest generation of GNSS receivers support most if not all of the GNSS constellation standards and frequencies, improving the accuracy and redundancy substantially. The incorporation of correction data from a fixed base station in close proximity to a GNSS receiver is a differential GNSS technique referred to as Real-Time Kinematic (RTK) [4]. The RTK GNSS capable receiver is typically referred to as the rover, with the RTK base station (a stationary GNSS receiver of accurately defined latitude, longitude and altitude) transmitting correction data using either radio systems or the internet. The RTK rover can achieve centimeter-level accuracy from an RTK base station in close proximity (typically within a 40 km radius) [5]. NTRIP [6] (Networked Transport of RTCM (Radio Technical Commission for Maritime Services) via Internet Protocol) is an open, non-proprietary protocol supporting all GNSS data formats [7], designed on the HTTP (HyperText Markup Language). The RTK base station transmits correction data at a frequency of 1 Hz using an NTRIP caster service over the internet to a NTRIP server (available in both open (e.g. RTK2go [8]) and commercial capacities). The position of the RTK base station can be determined with the addition of satellite phase bias information with a method termed Precise Point Positioning (PPP) [9]. The RINEX standard [10] is used to store positional and navigational data for this purpose, which include meteorological data and GNSS observations such as the code, phase, Doppler and time.

The development of low-cost geolocation alternatives opens up new possibilities and opportunities for research and commercial applications, in contrast to existing systems which are prohibitively expensive. This movement forms part of the *Civiltronics* [11] paradigm at the University of Pretoria, whereby core principles of traditional civil engineering is combined with computer science, information technology and electronic engineering, accelerating challenging research projects for even the most demanding applications [12], [13] which would otherwise not be affordable to implement [14]. Numerous other literature sources highlight these advantages, ranging from medicine [15], environmental engineering [16] to agricultural applications [17]. Sports engineering sees prominent applications of accurate geolocation services, ranging from sprint diagnostics [18] to roller skiing [19]. Estimation for satisfactory reliability and safety of autonomous vehicles specifies a 20 cm and 10 cm accuracy requirement for local and highway geometries, respectively [20]. Contradictory, these improved accuracies pose new design challenges for pavement structures engineered to take advantage of naturally wandering driving behavior [21] which will indirectly benefit from accurate geolocation services. Auxiliary geolocation data further improve photogrammetric reconstruction accuracies associated with ambiguous environmental geometry associated with railway environments [22], [23]. Wind-induced responses of buildings with amplitudes and natural frequencies exceeding 2 cm and 2 Hz, respectively, can be quantified with RTK-GNSS receivers [24].

PPP presents an improvement over the single-point positioning (SPP) technique [25]. PPP relies on carrier-phase measurements as the primary observable to model or estimate effects for centimetre-level resolution. Dual-frequency antennas and receivers are advantageous, eliminating ionospheric effects through the use of dual-frequency code and phase measurements. Tidal motion, satellite and antenna receiver offsets, carrier-phase windup, carrier phase ambiguities (resolved to integer values wherever possible) and residual tropospheric propagation delays are also modelled. Centimetre-level accuracies (1-sigma), resolved into their respective orthogonal axes, are achievable within 30 min, whereas millimetre-accuracy requires longer observation periods. PPP is vital for processing data from both static and kinematic applications, ranging from land surveying and mapping to real-time tsunami detection. RTK by comparison transmits carrier-phase and pseudorange data to local receivers using a specific communications medium. PPP and RTK together yield a high degree of accuracy and precision provided latency is maintained to a minimum, cycle slips are detected and corrected in real-time, and ambiguities are resolved promptly.

This paper presents the development, construction, configuration, deployment and verification of a low-cost, mobile RTK GNSS geolocation service developed at the Engineering 4.0 facility at the University of Pretoria, which consists of both a fixed RTK base station and an RTK rover. Whilst this implementation is not new [26], the miniaturised hardware and streamlined software significantly reduces the complexity associated with developing and operating such a system. The RTK implementation utilises commercially available components in a small form factor, providing a versatile and highly configurable solution for a range of research and testing applications, without the costs and limitations associated with comparable commercial offerings.

## 2 Hardware description

**Fig. 1:**
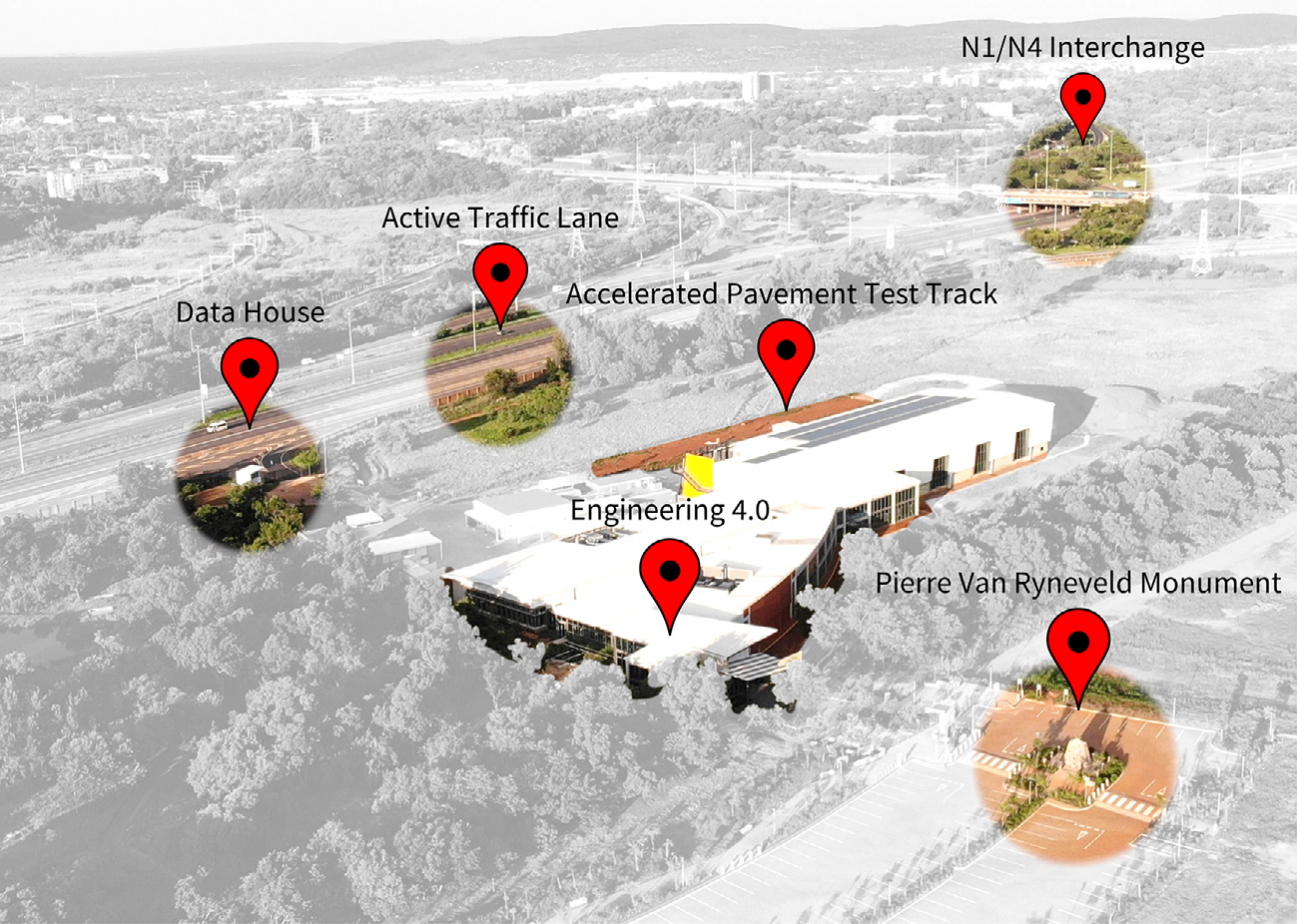
Aerial view of the Engineering 4.0 complex alongside key facilities.

Engineering 4.0 [1], commissioned on the 30th of November 2020, serves as the new research and training facility on the Hillcrest campus at the University of Pretoria (Fig. 1). As part of the research activities and projects associated with the new facility, the need for RTK-grade geolocation capabilities were identified subject to the following requirements:

• Blanket RTK coverage of the Hillcrest campus and surrounding region;
• Broadcasting of corrections using an internet connection;
• Power efficient design to minimise self-heating effects in the warm climate;
• High frequency (minimum of 1 Hz) acquisition capabilities for the RTK rover;
• Compatibility with open source/freeware configuration and caster software packages;
• Support for PPP geolocation of the base station antenna;
• Remote access control of the NTRIP caster service/server;
• Redundant power and communication systems;
• Small form factor to allow mobile deployment of both the RTK base station and RTK rover;
• Streamlined interface for ease of training and adoption in research projects, and
• Hardware support for all four of the primary GNSS constellations.

Even though National Geo-spatial Information (NGI) [27] manages TrigNet [28], a network of continuously operating GNSS base stations covering South Africa, the dynamic needs associated with research projects distributed over the country necessitates the development of RTK-related infrastructure. Research was carried out to determine the most cost-effective solution based on the list of requirements, commercially available hardware and software limitations. For both the RTK base station and RTK rover, the ZED-F9P GNSS module [29] manufactured by u-blox was selected as the receiver. The module supports RTK update frequencies ranging from 8 Hz (BeiDou, Galileo, GLONASS, GPS) to 20 Hz (GPS only), velocity and dynamic heading accuracies of 0.05 m/s and 0.3° respectively and a convergence time of less than 10 s. RTK performance is characterised by a circular error probable (CEP) to 10 mm + 1 ppm. The F9 engine supports a total of 184-channels (GPS L1C/A L2C, GLO L1OF L2OF, GAL E1B/C E5b, BDS B1I B2I, QZSS L1C/A L1S L2C and SBAS L1C/A).

### 2.1 RTK base station

Three software packages are required to transmit correction data for the RTK base station and to configure the receiver and PPP:

• SNIP [30] is an NTRIP caster application used to push data streams from one interface to another. The free (home and commercial use) Lite version supports up to three data streams. SNIP supports both Linux and Windows operating systems (OS);
• u-center [31] is the evaluation and configuration software package developed by u-blox for all u-blox GNSS receivers. u-center support is restricted to Windows OS, and
• RTKLIB [32] (shorthand for “RTK library”) is a software suite used for RTK applications [33]. The RTK2CONV of the software suit is used for generating the required RINEX observation files for PPP. RTKLIB supports Windows OS with limited Linux support.

**Fig. 2:**
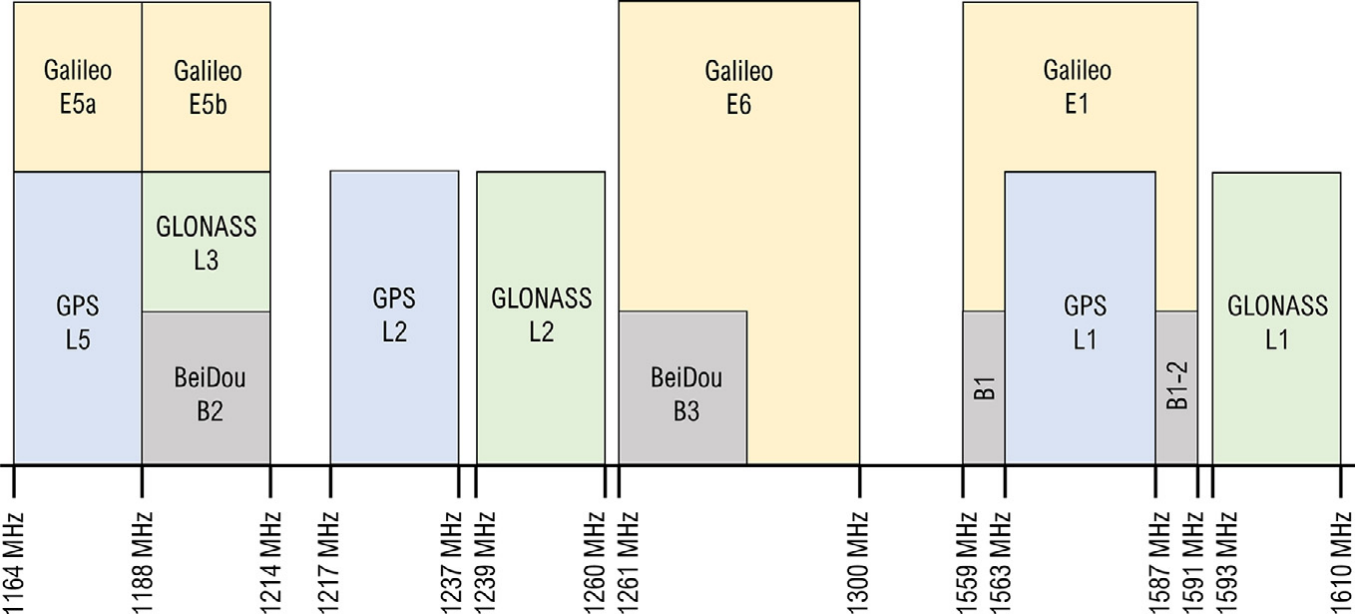
GNSS signal frequency bands used by various operators (adapted from [3]).

With u-center limiting the selection of the OS to Windows, a low-power LattePanda 4 Gb/64 Gb microcomputer [34] was selected for the RTK base station. The LattePanda is installed within a transparent enclosure together with passthrough cables for peripherals, including an RF antenna connector. The GNSS receiver, in the form of a breakout board manufactured by SparkFun [35], is connected to the LattePanda using a USB-C cable. The small footprint of the hardware installed in a rigid enclosure enables easy relocation for field experimentation. The IP-67 rated Tallysman TW1829 dual-band antenna [36], [3] supports GPS/QZSS L1 (1575.42 MHz) and L2 (1227.6 MHz), GLONASS G1 (1602 MHz) and G2 (1248 MHz), Galileo E1 (1575.42 MHz) and BeiDou B1 (1575.42 MHz) frequencies (Fig. 2), serving as a cost-effective antenna for the GNSS receiver module. The antenna design features a tuned, circular dual feed, stacked patch element with an integrated, dual-stage wide-band LNA (low noise amplifier).

### 2.2 RTK rover

**Fig. 3:**
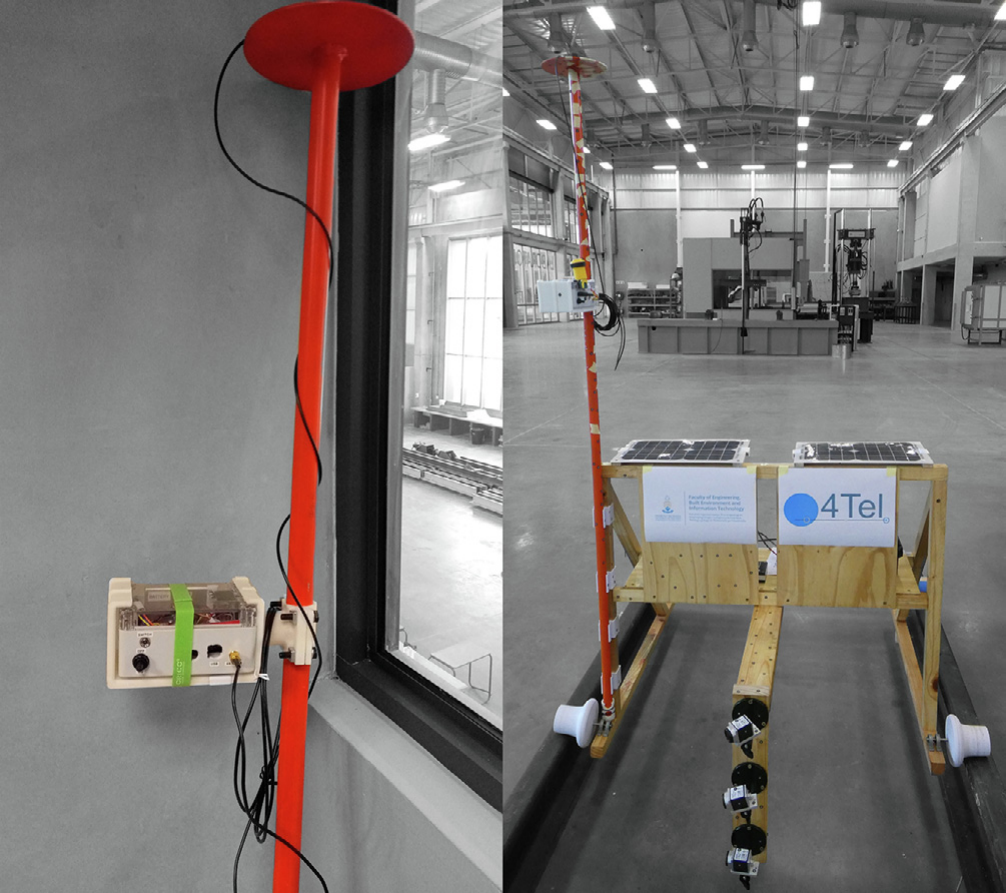
Prism pole with RTK rover attached for two different configurations.

For the RTK rover, a small form factor was adopted for added mobility. An Arduino-based microcontroller (SparkFun SAMD51 Thing Plus [37]) connects to the u-blox ZED-F9P breakout using the I2C interface, along with a high-accuracy inertial measurement unit (IMU), button detector, OLED display and SD card reader. The rechargeable 2 000 mAh Lithium Polymer battery provides approximately 4 h of runtime for the RTK rover. The button detector is connected to a 3.5 mm audio connector on the side of the enclosure, providing easy installation of an external trigger for recording data to the SD card. Alternatively, the addition of the microcontroller allows for parsed GNSS data to be sent over the USB cable as a serial interface to another computer. The enclosure can be readily installed on a rigid prism pole to serve as a low-cost surveying solution, either hand-operated (Fig. 3, left) or fixed to another vehicle (Fig. 3, right).

The proposed geolocation service demonstrates the following advantages which are applicable to the wider user community:

• Costs associated with the hardware are orders of magnitude smaller than commercially available solutions and is not restricted to the closed ecosystem of a commercial manufacturer;
• Deployment of RTK capabilities over a wide geographic region where there is limited or no existing coverage, especially in remote areas where research is typically conducted;
• The mobility presented by the rover opens up a number of potential applications, ranging from the surveying of civil infrastructure such as catchments and hydrological structures, geotagging of vegetation in remote areas, sports engineering [18], [19], monitoring of earth movements and settlement associated with large geotechnical structures [38], dynamic response of structures [24], autonomous navigation platforms [2], [20], [21] - to the digitisation of historical structures and measurement of road geometry - as presented in this article;
• Customisability of the RTK rover, for example, adding a spectrometer or PH sensor for geotagging biological and chemical signatures of fresh produce and soil in the field, and
• Educating students on state-of-the-art surveying and measurement techniques [11].

## 3 Design files

The complete list of design files is summarised in Table 1. These files provide the necessary information and software to duplicate and implement an equivalent RTK geolocation service, for both the RTK base station and RTK rover. The files are accessible from the Open Science Framework source file repository specified within this manuscript.

**Table 1:** Complete list of design files.

Design file name	File type	Open source license	Location of the file
RTK_Caster.ino	Arduino source code (.ino)	CC BY 4.0	Source file repository (Arduino\RTK_Caster)
RTK_Rover.ino	Arduino source code (.ino)	CC BY 4.0	Source file repository (Arduino\RTK_Rover)
CC_combined.bin	CloudCompare (.bin)	CC BY 4.0	Source file repository (Experiments\Monument)
Hovermap.laz	Lidar scan data (.laz)	CC BY 4.0	Source file repository (Experiments\Monument)
Recap_Photogrammetry.obj	3D geometry data (.obj)	CC BY 4.0	Source file repository (Experiments\Monument)
RTK_GPS.csv	Text file (.csv)	CC BY 4.0	Source file repository (Experiments\Monument)
Hillcrest_campus.csv	Text file (.csv)	CC BY 4.0	Source file repository (Experiments\RTK_Coverage)
Pretoria_East.csv	Text file (.csv)	CC BY 4.0	Source file repository (Experiments\RTK_Coverage)
GPS_golfcart.csv	Text file (.csv)	CC BY 4.0	Source file repository (Experiments\Speed_Arrestor)
GPS_ground.csv	Text file (.csv)	CC BY 4.0	Source file repository (Experiments\Speed_Arrestor)
Hovermap_LiDAR_subset.csv	Text file (.csv)	CC BY 4.0	Source file repository (Experiments\Speed_Arrestor)
main.py	Python script (.py)	CC BY 4.0	Source file repository (Experiments\Speed_Arrestor)
Track_Level.xlsx	Excel file (.xlsx)	CC BY 4.0	Source file repository (Experiments\Level)
Track_RTKGPS.xlsx	Excel file (.xlsx)	CC BY 4.0	Source file repository (Experiments\Level)
210318_194209.ubx	Proprietary u-blox (.ubx)	CC BY 4.0	Source file repository (PPP)
210318_194209.obs	RINEX observations (.obs)	CC BY 4.0	Source file repository (PPP)
210318_194209.pdf	PDF	CC BY 4.0	Source file repository (PPP)
Caster.pdf	PDF	CC BY 4.0	Source file repository (Schematics folder)
Rover.pdf	PDF	CC BY 4.0	Source file repository (Schematics folder)
Part 1 - Surveying PPP.mp4	Video file (MP4)	CC BY 4.0	Source file repository (Videos)
Part 2 - NTRIP Caster.mp4	Video file (MP4)	CC BY 4.0	Source file repository (Videos)
Part 3 - NTRIP Client.mp4	Video file (MP4)	CC BY 4.0	Source file repository (Videos)

A description of each design file follows:

*GNSS_Caster.ino*: Arduino source code to display the uptime on an OLED screen, installed within the RTK base station. The OLED is driven by the SparkFun Blackboard C board over the I2C interface.

*GNSS_Rover.ino*: Arduino source code for the RTK rover. Recording of the data to the SD card is triggered by an external button connected to the 3.5 mm audio connector. The I2C frequency is configured for 150 kHz which represents the maximum stable frequency, limited by the OLED display.

*CC_combined.bin*: CloudCompare binary file for the LiDAR, photogrammetry and RTK-ASM point clouds (refer to the Validations and Characterization section).

*Hovermap.laz*: LiDAR point cloud data obtained from the Hovermap scan for the monument (refer to the Validations and Characterization section).

*Recap_Photogrammetry.obj*: Object model for the aerial photogrammetric reconstruction of the monument (refer to the Validations and Characterization section).

*RTK_GPS.csv*: Raw data for the RTK measurements of the monument (refer to the Validations and Characterization section).

*Hillcrest_campus.csv*: RTK coverage mapping data for Hillcrest campus (refer to the Validations and Characterization section).

*Pretoria_East.csv*: RTK coverage mapping data for the Eastern region of Pretoria (refer to the Validations and Characterization section).

*GPS_golfcart.csv*: RTK rover speed arrestor geometry measurements using the golfcart (refer to the Validations and Characterization section).

*GPS_ground.csv*: RTK rover ground truth measurements (refer to the Validations and Characterization section).

*Hovermap_LiDAR_subset.csv*: Sample of the Hovermap point cloud associated with the speed arrestor (refer to the Validations and Characterization section).

*Track_Level.xlsx*: Digital Level measurements of the PY Slab Track (refer to the Validations and Characterization section).

*Track_RTKGPS.xlsx*: RTK GPS measurements of the PY Slab Track (refer to the Validations and Characterization section).

*Speed_Arrestor/main.py*: Python script used to graph the point cloud data as illustrated in Fig. 24 (refer to the Validations and Characterization section).

*210318_194209.ubx*: Raw observation data recorded using u-center as part of the PPP of the antenna (data recorded on 18 and 19 March 2021).

*210318_194209.obs*: RINEX observation data obtained from the raw observation data, converted using RTK2Lib’s rtkconv.exe utility.

*210318_194209.pdf*: Processed RINEX report illustrating the coordinates, measurement errors and various other information from the Canadian Spatial Reference System (CRPS) [51].

*Caster.pdf*: Electronic schematic detailing the hardware configuration of the RTK base station.

*Rover.pdf*: Electronic schematic detailing the hardware configuration of the RTK rover.

*Part 1 – Surveying PPP.mp4*: Detailed video tutorial [50] to survey in the antenna using both a fast-averaging method and more precise PPP with u-center and CRPS [51] respectively.

*Part 2 – NTRIP Caster.mp4*: Detailed video tutorial [52] to correctly configure the RTK base station’s GNSS receiver to operate in RTK mode and instructions on configuring SNIP and RTK2go to transmit the correction data to the NTRIP server.

*Part 3 – NTRIP Client.mp4*: Detailed video tutorial [53] to correctly configure the RTK rover’s GNSS receiver to operate as an NTRIP client to incorporate correction data, providing RTK measurements.

## 4 Bill of materials

**Fig. 4:**
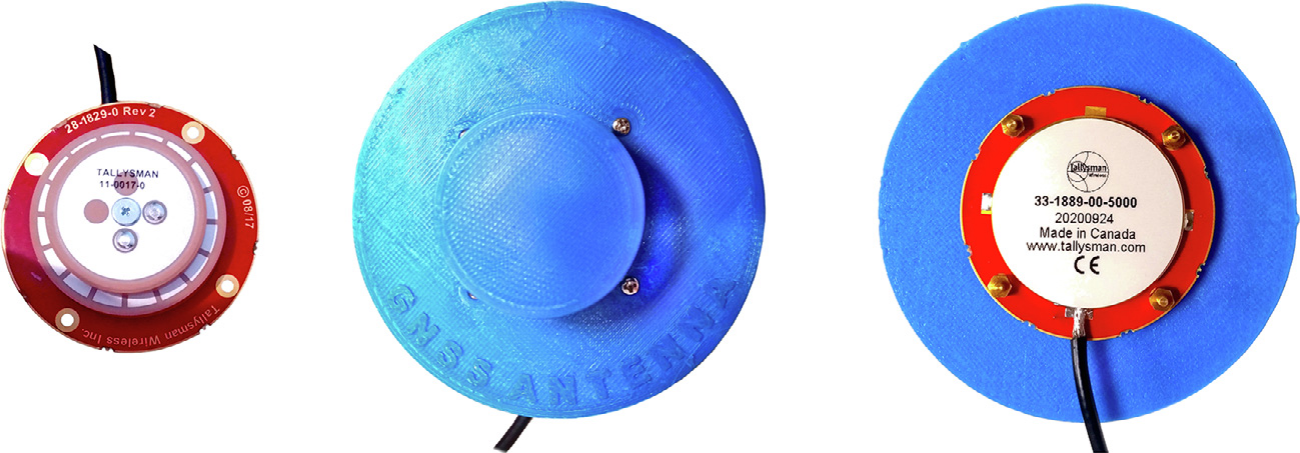
Protective GNSS antenna cover providing additional weather- and UV-resistance.

The complete bill of materials (BOM) to replicate the RTK base station and RTK rover is listed in Table 2, Table 3 respectively. The listed components are not specialised and can be sourced online from several local (South African) and international suppliers (RS, SparkFun). The 3D printed mounting bracket developed for the RTK rover (Fig. 3) and sacrificial UV-cover for the base station antenna (Fig. 4) are excluded from the BOM.

**Table 2:** RTK rover Bill of Materials.

Designator	Component	Number	Cost per unit - currency	Total cost - currency	Source of materials	Material type
Primary components	SparkFun Qwiic Button Breakout BOB-15931	1	$2.95 USD	$2.95 USD	SparkFun	Other
	SparkFun GPS-RTK-SMA Breakout - ZED-F9P (Qwiic) GPS-16481	1	$219.95 USD	$219.95 USD	SparkFun	Other
	Concave Button - Yellow COM-09338	1	$2.50 USD	$2.50 USD	SparkFun	Other
	SparkFun VR IMU Breakout - BNO080 (Qwiic) SEN-14686	1	$34.95 USD	$34.95 USD	SparkFun	Other
	SparkFun Bluetooth Mate Gold WRL-12580	1	$36.95 USD	$36.95 USD	SparkFun	Other
	SparkFun Level Shifting microSD Breakout DEV-13743	1	$5.50 USD	$5.50 USD	SparkFun	Other
	Battery Li-Po 2000mAh 3.7 V DTP605068	1	$10.38 USD	$10.38 USD	SparkFun	Other
	SparkFun Thing Plus - SAMD51 DEV-14713	1	$19.95 USD	$19.95 USD	SparkFun	Other
Antenna	GNSS antenna TALLYSMAN GPS L1/L2, GLONASS G1/G2/G3, GALILEO E1/E5b, BEIDOU B1/B2, ANTENNA, 26 dB, 5 M RG174, SMA-M 33–1889-00–5000	1	$131.42 USD	$131.42 USD	RF Design	Other
Enclosure	Enclosure IP65 − 115 X 90 X 55 - Beige - Clear Gainta Enclosures Germany (G212C)	1	$13.17 USD	$13.17 USD	MicroRobotics	Other
Panel mounts	SMA passthrough RS PRO Black RF Coaxial Cable 50 Ω 7,942,923	1	$13.69 USD	$13.69 USD	RS	Other
Cables	Qwiic Cable - Female Jumper (4-pin) CAB-14988	1	$1.50 USD	$1.50 USD	SparkFun	Other
	Qwiic Cable − 100 mm PRT-14427	1	$1.50 USD	$1.50 USD	SparkFun	Other
	Qwiic Cable − 50 mm PRT-14426	4	$0.95 USD	$3.80 USD	SparkFun	Other
	Qwiic Multiport SPX-16906	1	$1.95 USD	$1.95 USD	SparkFun	Other
	USB to Micro USB Fast − 1 m Cable LS63S	1	$4.53 USD	$4.53 USD	MicroRobotics	Other
Electronic components	OLED 0.96 in. Display I2C White OLED096W	1	$6.83 USD	$6.83 USD	MicroRobotics	Other
	Resistors 1/4″ (33kΩ)	2	$0.10 USD	$0.20 USD	Generic	Other
	Switch Toggle Switch 2 Pin - Splash Proof (4 Pack) MTS-101-COVER	1	$2.02 USD	$2.02 USD	MicroRobotics	Other
	3.5 mm female socket	1	$1.00 USD	$1.00 USD	Generic	Other
	microSD Card − 16 GB (Class 10) COM-14832	1	$19.95 USD	$19.95 USD	SparkFun	Other
	Standoffs Hex 2.5 mm 6X6mm Kit - (10 Pack) M25-66-HEX	1	$1.05 USD	$1.05 USD	MicroRobotics	Other
	Standoffs for PCB 10 mm (10 Pack) FIT0066	1	$2.09 USD	$2.09 USD	MicroRobotics	Other
	Starrett 42 mm Spirit Level 0,432,536	1	$4.32 USD	$4.32 USD	RS	Other
	Insulation tape	1	$1.00 USD	$1.00 USD	Generic	Other
	Hi-Bond Transparent VST 4100CP Double Sided Adhesive Square, 30 mm × 30 mm 6,861,141	1	$3.27 USD	$3.27 USD	RS	Other
	Concave Button - Yellow COM-09338	1	$2.50 USD	$2.50 USD	SparkFun	Other
Accessories	Jumpers (F/F), Veroboard, female and male headers					Other
	**Total cost of the RTK rover**			**$548.92 USD**

**Table 3:** RTK base station Bill of Materials.

Designator	Component	Number	Cost per unit - currency	Total cost - currency	Source of materials	Material type
Primary hardware	LattePanda 4G/64 GB Support Windows 10 DFR0419	1	$194.38 USD	$194.38 USD	MicroRobotics	Other
	SiPy – Sigfox, Wi-Fi and BLE AF3534	1	$50.81 USD	$50.81 USD	MicroRobotics	Other
	Pysense - Sensor Breakout AF3507	1	$47.74 USD	$47.74 USD	MicroRobotics	Other
	SparkFun BlackBoard C SPX-16282	1	$14.95 USD	$14.95 USD	SparkFun	Other
	LattePanda acrylic case FIT0474	1	$6.62 USD	$6.62 USD	MicroRobotics	Other
	Aluminum Heatsink + Fan for LattePanda	1	$13.80 USD	$13.80 USD	MicroRobotics	Other
Antennas	Antenna Kit 900Mhz, for LoPy, LoRa, SiPy AF3340	1	$6.83 USD	$6.83 USD	MicroRobotics	Other
	Antenna 2.4 GHz Wireless 2DBI BPI-2DB	1	$2.65 USD	$2.65 USD	MicroRobotics	Other
	GNSS antenna TALLYSMAN GPS L1/L2, GLONASS G1/G2/G3, GALILEO E1/E5b, BEIDOU B1/B2, ANTENNA, 26 dB, 5 M RG174, SMA-M 33–1889-00–5000	1	$131.42 USD	$131.42 USD	RF Design	Other
Enclosure	Schneider Electric Thalassa PLM, Polyester Wall Box, IP66, 160 mm × 310 mm × 215 mm	1	$122.73 USD	$122.73 USD	RS	Other
Peripherals	HP P204 19.5-inch Monitor (HDMI) 5RD65AS	1	$62.38 USD	$62.38 USD	MicroRobotics	Other
	K22 Slimline Wireless 2.4 GHz Keyboard + Large Touch ZW-K22	1	$32.41 USD	$32.41 USD	MicroRobotics	Other
Panel mounts	Panel Mount USB-C Extension Cable − 6″ CAB-15455	1	$7.95 USD	$7.95 USD	SparkFun	Other
	Panel Mount USB Micro-B Extension Cable − 6″ CAB-15464	1	$2.50 USD	$2.50 USD	SparkFun	Other
	Panel Mount HDMI Male to Female AF978	1	$4.32 USD	$4.32 USD	MicroRobotics	Other
	Ehternet passthrough AF909	1	$4.11 USD	$4.11 USD	MicroRobotics	Other
	Panel Mount USB Cable - A Male to A Female AF908	1	$5.92 USD	$5.92 USD	MicroRobotics	Other
	SMA passthrough RS PRO Black RF Coaxial Cable 50 Ω 7,942,923	1	$13.69 USD	$13.69 USD	RS	Other
	USB Hub 4 Port + Switch USBHUB4	1	$4.32 USD	$4.32 USD	MicroRobotics	Other
Cables	USB Mini-B Cable − 6″ CAB-13243	1	$1.95 USD	$1.95 USD	SparkFun	Other
	Reversible USB A to C Cable − 0.3 *m* CAB-15426	1	$3.95 USD	$3.95 USD	SparkFun	Other
	USB 2.0 Cable A to C − 3 Foot CAB-15092 ROHS	1	$3.95 USD	$3.95 USD	SparkFun	Other
	USB to Micro USB Fast − 1 m Cable LS63S	1	$4.53 USD	$4.53 USD	MicroRobotics	Other
	HDMI Cable 1.5 M HDMI-1.5 M	1	$4.81 USD	$4.81 USD	MicroRobotics	Other
Electronic components	Qwiic Cable − 100 mm PRT-14427	1	$1.50 USD	$1.50 USD	SparkFun	Other
	OLED 0.96 in. Display I2C White OLED096W	1	$6.83 USD	$6.83 USD	MicroRobotics	Other
	LED Light Bar - White (SMD) COM-12014	1	$4.95 USD	$4.95 USD	SparkFun	Other
	12 V Step-Up Voltage Regulator U3V12F12 2117	1	$5.51 USD	$5.51 USD	MicroRobotics	Other
	Hi-Bond Transparent VST 4100CP Double Sided Adhesive Square, 30 mm × 30 mm 6,861,141	1	$3.27 USD	$3.27 USD	RS	Other
	Resistors 1/4″ (4 7 0)	2	$0.10 USD	$0.20 USD	Generic	Other
	LED Red 3 mm (10 Pack) LED002	1	$0.70 USD	$0.70 USD	MicroRobotics	Other
	LED Holder 3 mm Black (10 Pack) LEDH-03-BLK	1	$1.32 USD	$1.32 USD	MicroRobotics	Other
	Capacitor 1000uF 35 V (4 pack) 1000UF-35 V-4	1	$0.84 USD	$0.84 USD	MicroRobotics	Other
	Screw Terminal Block 2 Pin − 2.54 mm (10 Pack)	1	$1.74 USD	$1.74 USD	MicroRobotics	Other
Power	Uninterruptable power supply (UPS) AP SERIES 720VA UPS	1	$34.50 USD	$34.50 USD	PSS	Other
	5 V 3A USB PSU − 1 × USB Port 5V3A-USB	2	$5.92 USD	$11.84 USD	MicroRobotics	Other
Software	Window 10 Home License Key	1	$160 USD	$160 USD	Windows Store	Other
Accessories	Veroboard, jumpers (M/M), female headers, glue gun					Other
	**Total cost of the RTK base station**			**$981.92 USD**

## 5 Building instructions

The building instructions are sub-divided into a short summary for both the RTK base station and RTK rover.

### 5.1 RTK base station

**Fig. 5:**
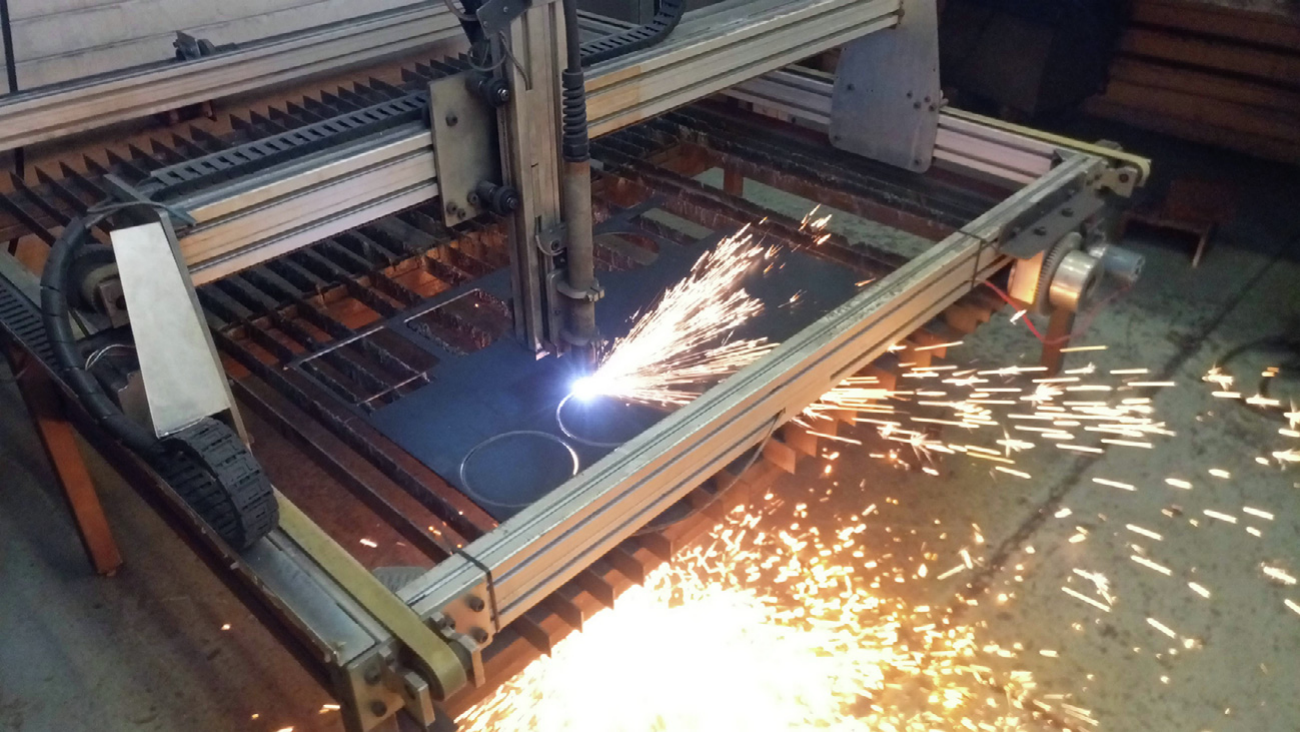
Antenna ground plane fabrication using a CNC plasma cutter.

The RTK base station hardware was installed at the Data House (Fig. 1), a dedicated facility adjacent to the N4 freeway for transportation research. The location provides an unobstructed view of the horizon, maximizing the number of satellites within line of sight of the antenna. A 200 mm diameter ground plane serves to mitigate unwanted multipath effects [39]; the ground planes (for both the RTK base station and RTK rover antennas) were fabricated from a 3 mm mild steel plate using a CNC plasma cutter (Fig. 5).

**Fig. 6:**
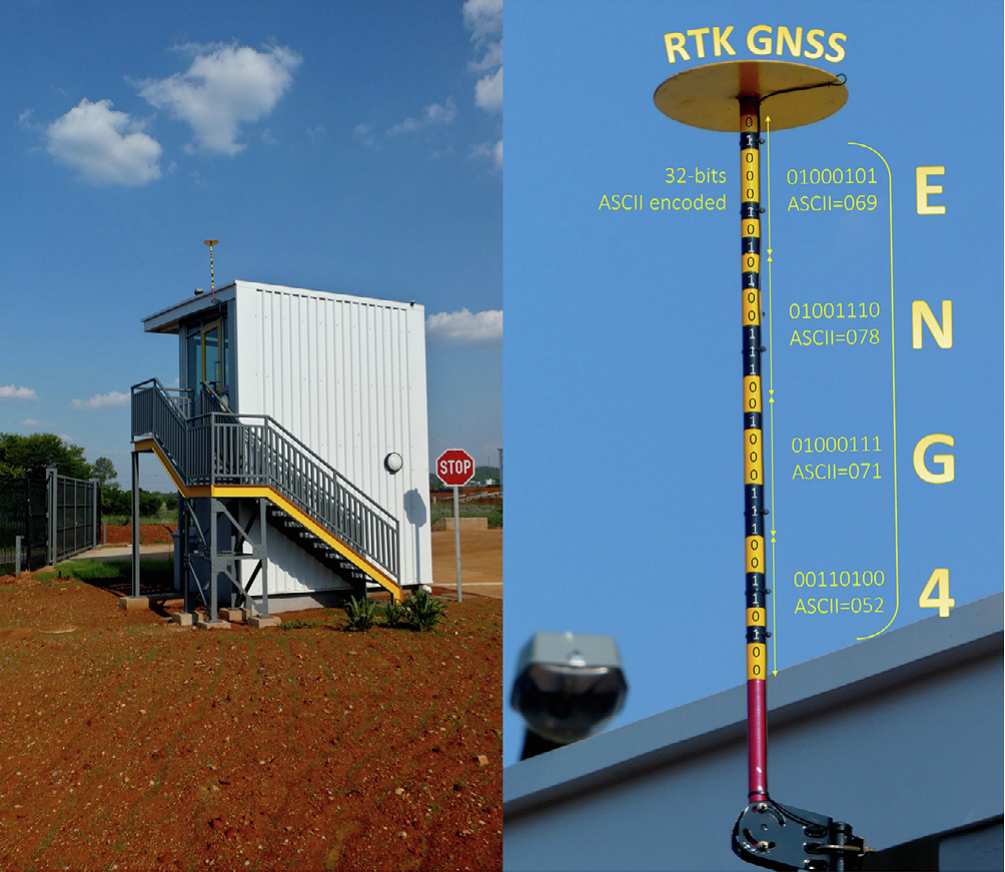
Data house (left) with the GNSS antenna installation (right).

The GNSS antenna was fixed to the ground plane using standoff screws, with the 5 m length of cable passing through a sealed enclosure to the interior of the Data House. Electrical power - which includes an automatic backup generator in addition so a dedicated uninterruptable power supply (UPS) - and high-speed internet connection is connected to the RKT base station in the interior of the Data House (See Fig. 6).

**Fig. 7:**
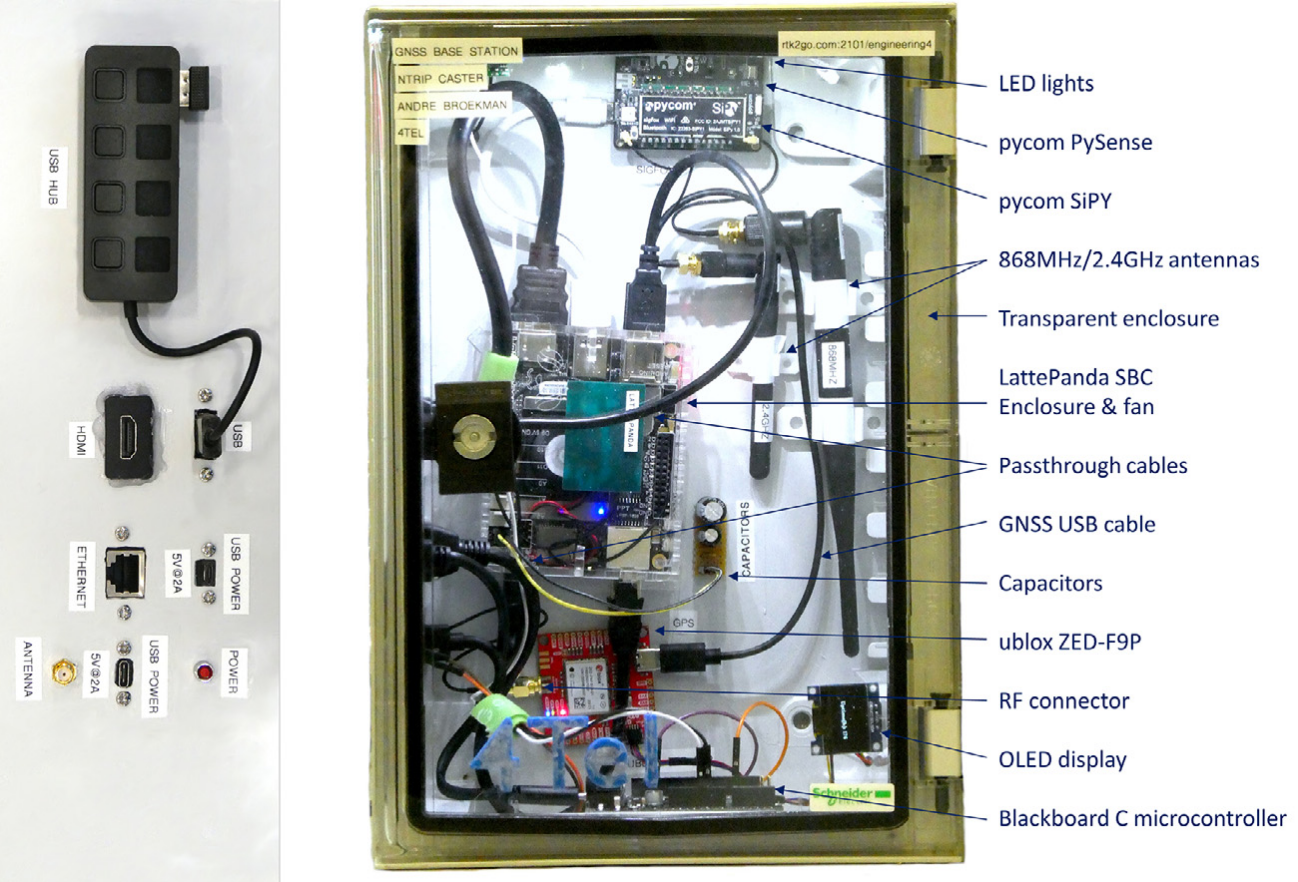
Annotated side (left) and front (right) view of the RTK base station.

**Fig. 8:**
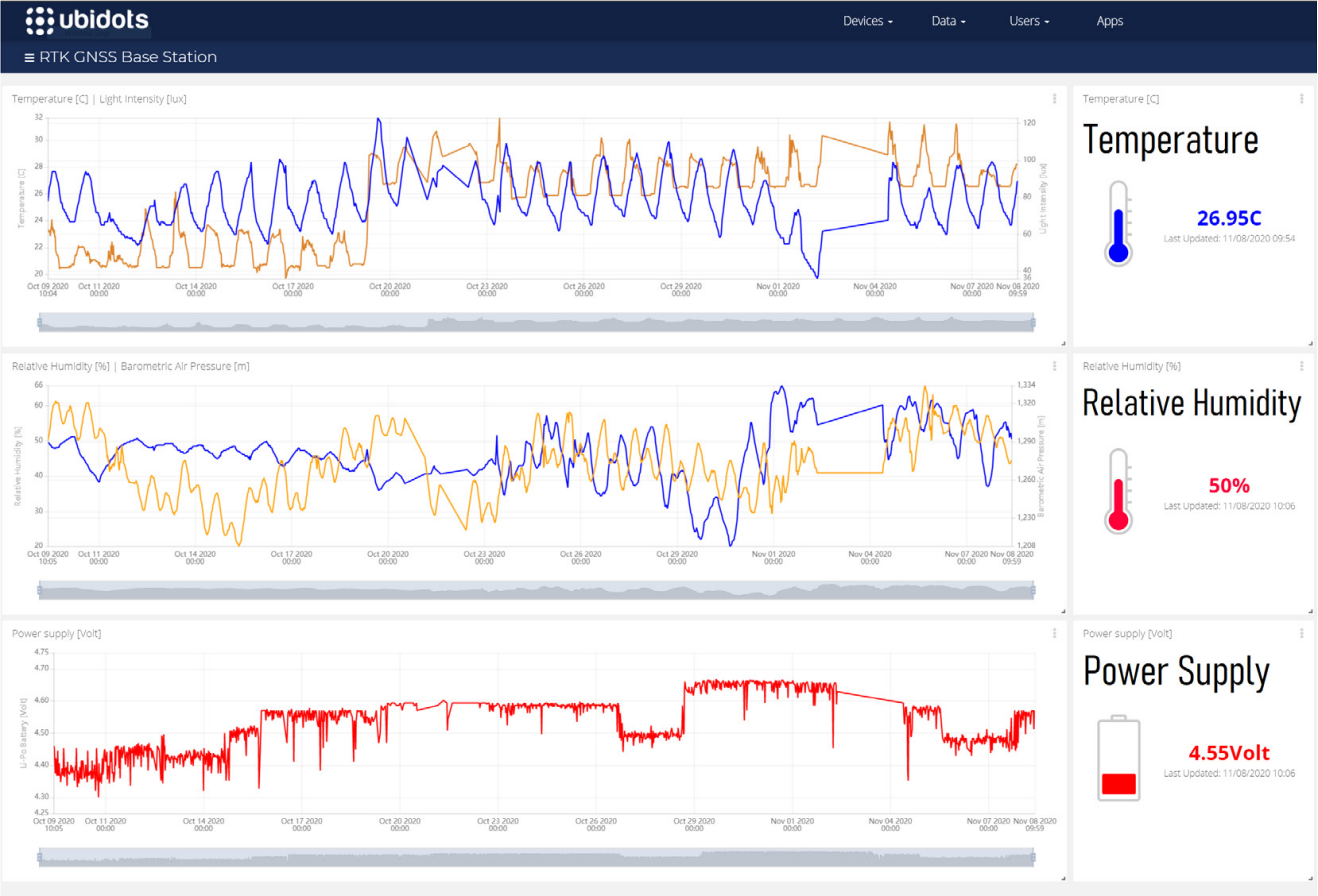
Dashboard illustrating environmental measurements of the RTK base station.

**Fig. 9:**
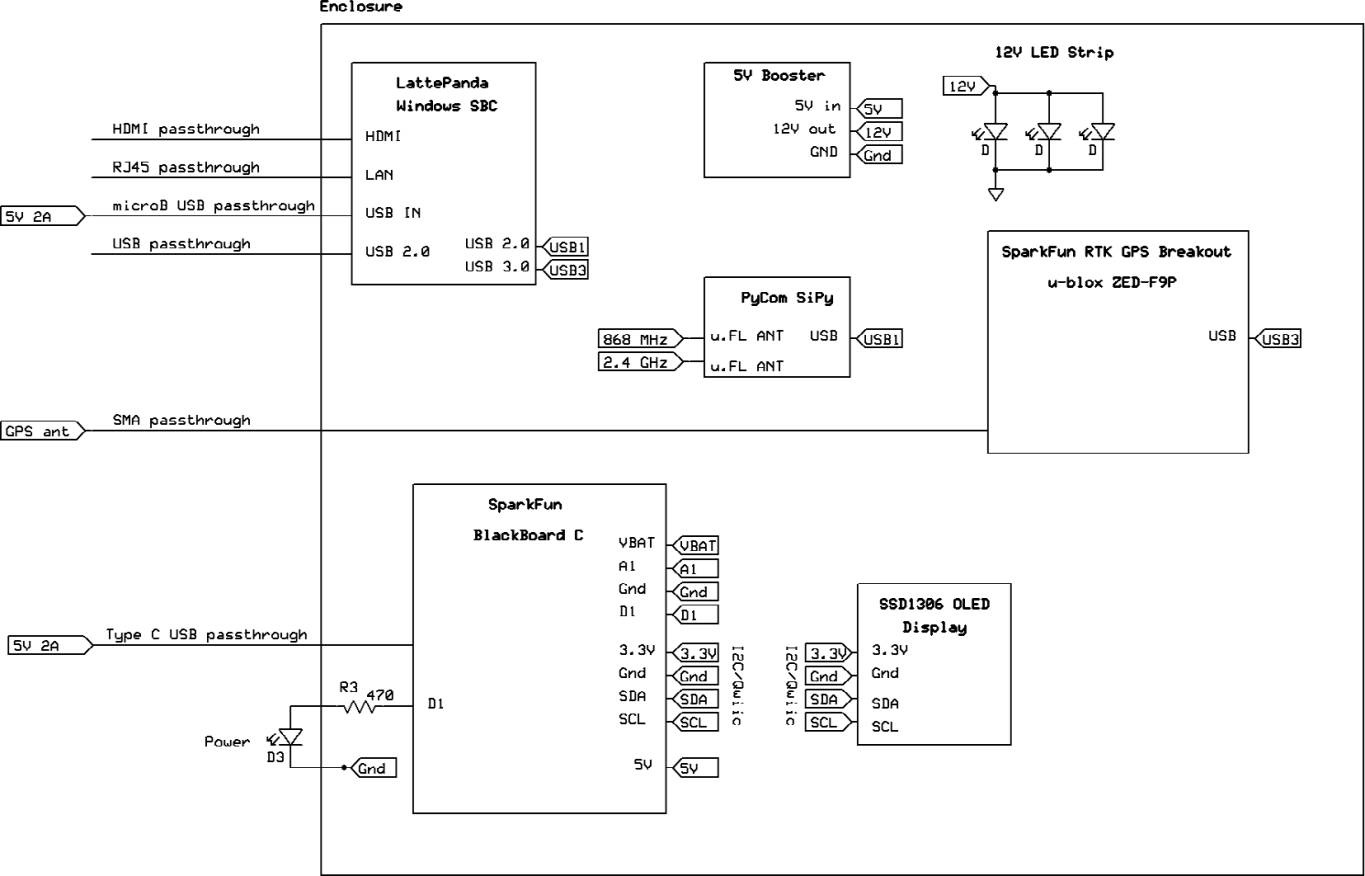
Electronic design of the RTK base station.

Fig. 7 illustrates the peripheral connections (USB hub, HDMI, Ethernet, USB input power (×2), RF connector and status LED) along with the hardware components through the transparent door of the RTK base station enclosure. The LED strip installed on the top face of the enclosure provides clear illumination of the labelled components. All the components are held in place using adhesive pads, allowing easy modification and replacement of components, which proved necessary as part of the testing phases. A Pycom SiPy microcontroller [40] with an environmental sensor expansion board (PySense) [41] is included, transmitting the internal temperature and other measurements (using SigFox) to the centralised Innovation Africa data platform [42] (Fig. 8). The power efficient design did not significantly increase the internal temperature of the enclosure, although a small exhaust fan is recommended for warmer operating environments. Fig. 9 illustrates the electronic design of the RTK base station with the primary components and connections among them.

### 5.2 RTK rover

**Fig. 10:**
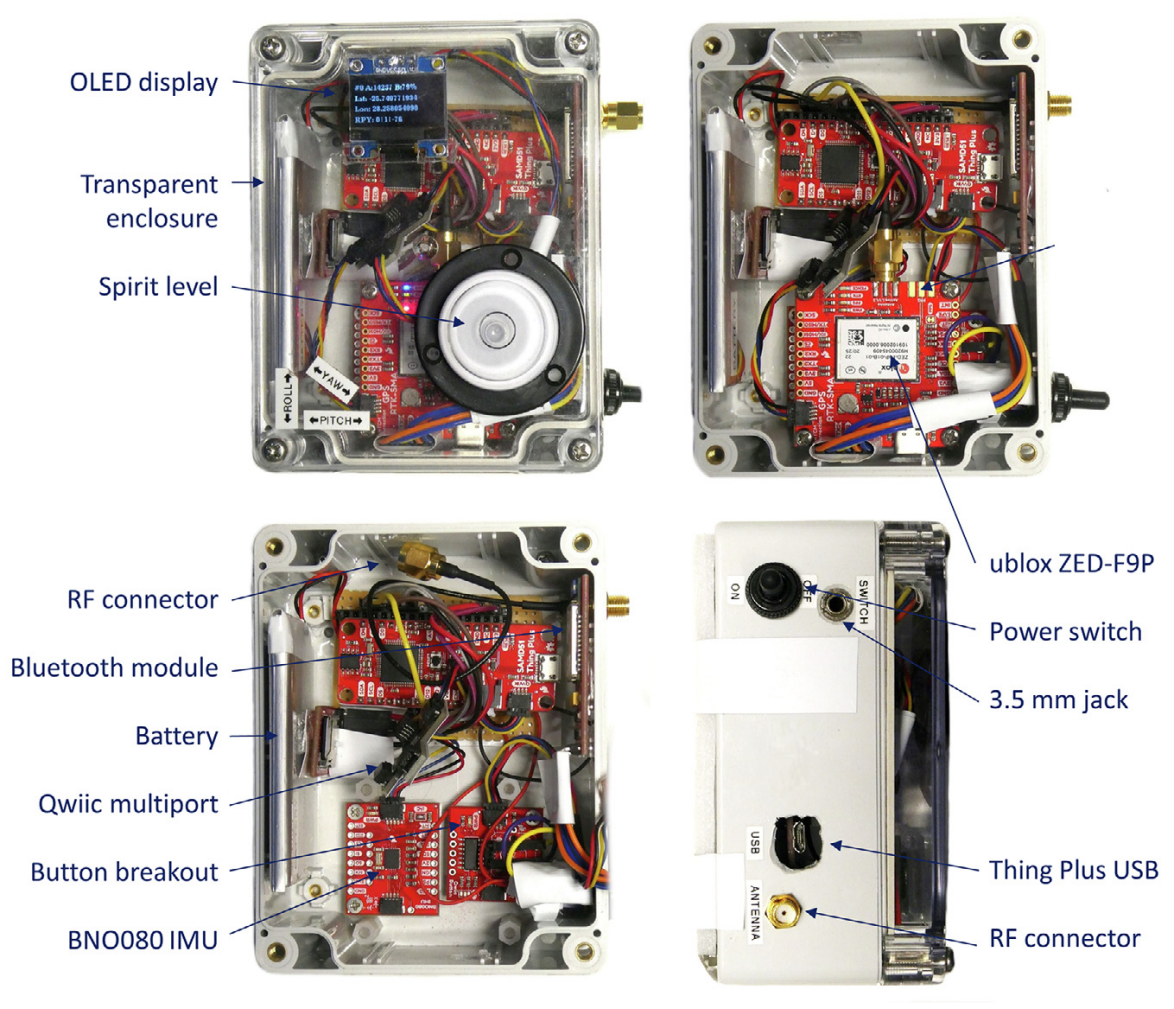
Annotated illustration of the RTK rover (top-left) with the cover (top-right) and GNSS breakout removed (bottom-left) alongside a side view (bottom-right).

**Fig. 11:**
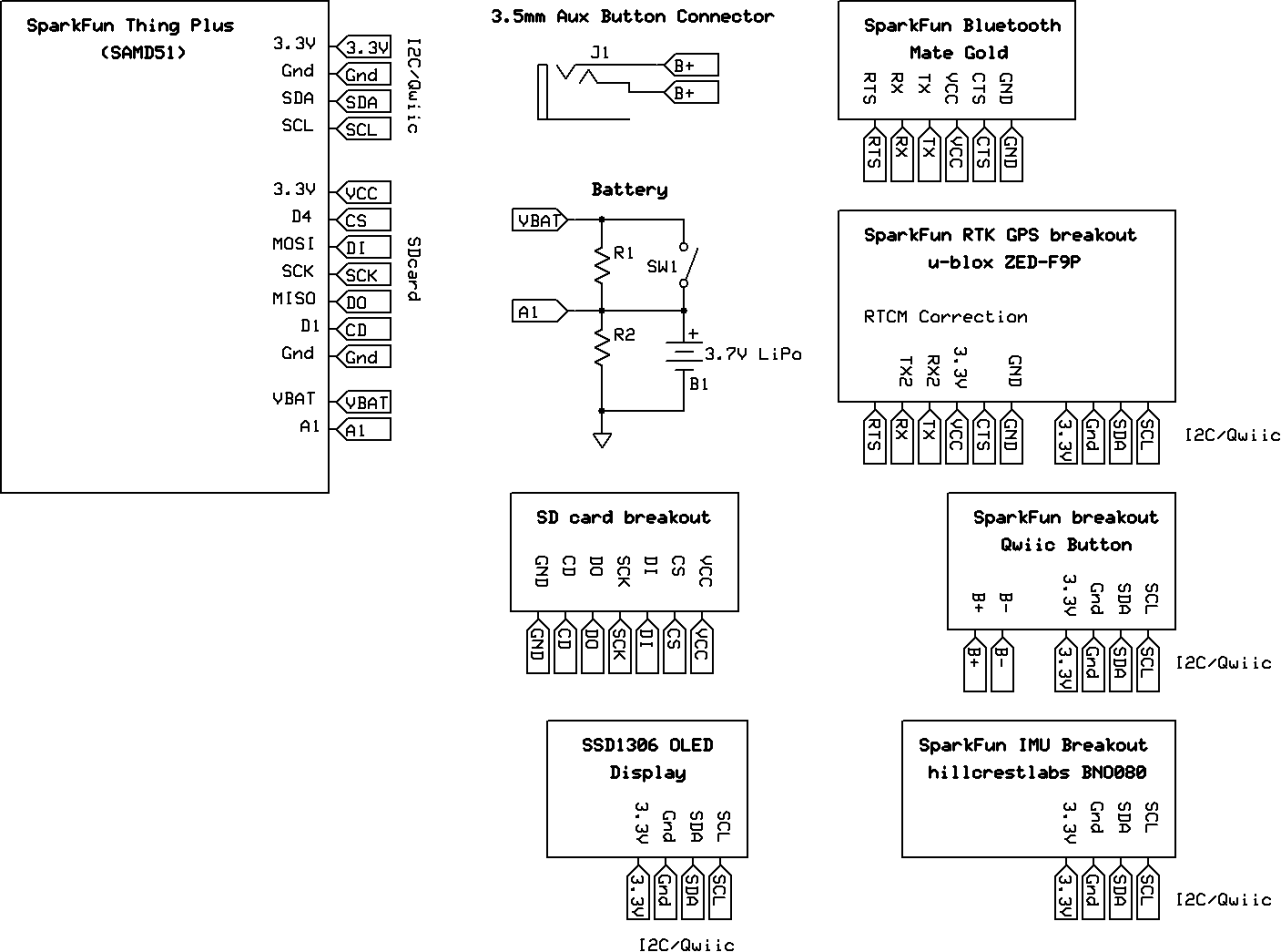
Electronic design of the RTK rover.

The RTK rover is illustrated in Fig. 10. The transparent cover of the enclosure allows for the OLED display to be visible through the acrylic along with the other hardware components and status LEDs. With the GNSS receiver removed (Fig. 10, bottom-left), the IMU and button breakout are revealed underneath. The IMU is aligned orthogonally with the enclosure, allowing any antenna offsets or rotation of the frame or prism pole to be corrected using simple Euclidean algebra. The GNSS receiver passthrough RF connector (SMA) is visible on the side of the enclosure along with the microcontroller’s USB port, power switch and the 3.5 mm audio connector for the external trigger. A thin layer of foam is glued to the bottom of the enclosure, ensuring a conformal fit with the 3D printed mounting bracket. The introduction of the Qwiic I2C interconnect system by SparkFun saves a significant amount of space and wiring among the breakouts. Jumper wires connect the Bluetooth module to the RTK UART (Universal Asynchronous Receiver/Transmitter) interface of the GNSS receiver breakout to pass through correction data. The battery is recharged automatically using the microcontroller’s built-in battery charger when connected with a USB cable. Fig. 11 illustrates the simplicity of the electronic design of the RTK rover. The final geolocation information from the GPS received is also communicated over the I2C protocol and is simultaneously stored on the non-volatile storage medium (SD card) and sent through the microcontroller’s USB Serial interface.

## 6 Operation instructions

The operating instructions are sub-divided into six sections:

• Software installation and system settings;
• Surveying of the antenna (averaging and PPP);
• Configuration of the RTK base station and NTRIP server for RTK operation;
• Configuration of the RTK rover for RTK operation;
• NTRIP client software configuration, and
• Operation modes of the RTK rover for data collection.

Extensive reference was made to detailed information provided by rtklibexplorer [43], Sky Horse Tech [44] and SparkFun [45], [46] to develop the RTK implementation presented in this chapter.

### 6.1 Software installation and system settings

**Fig. 12:**
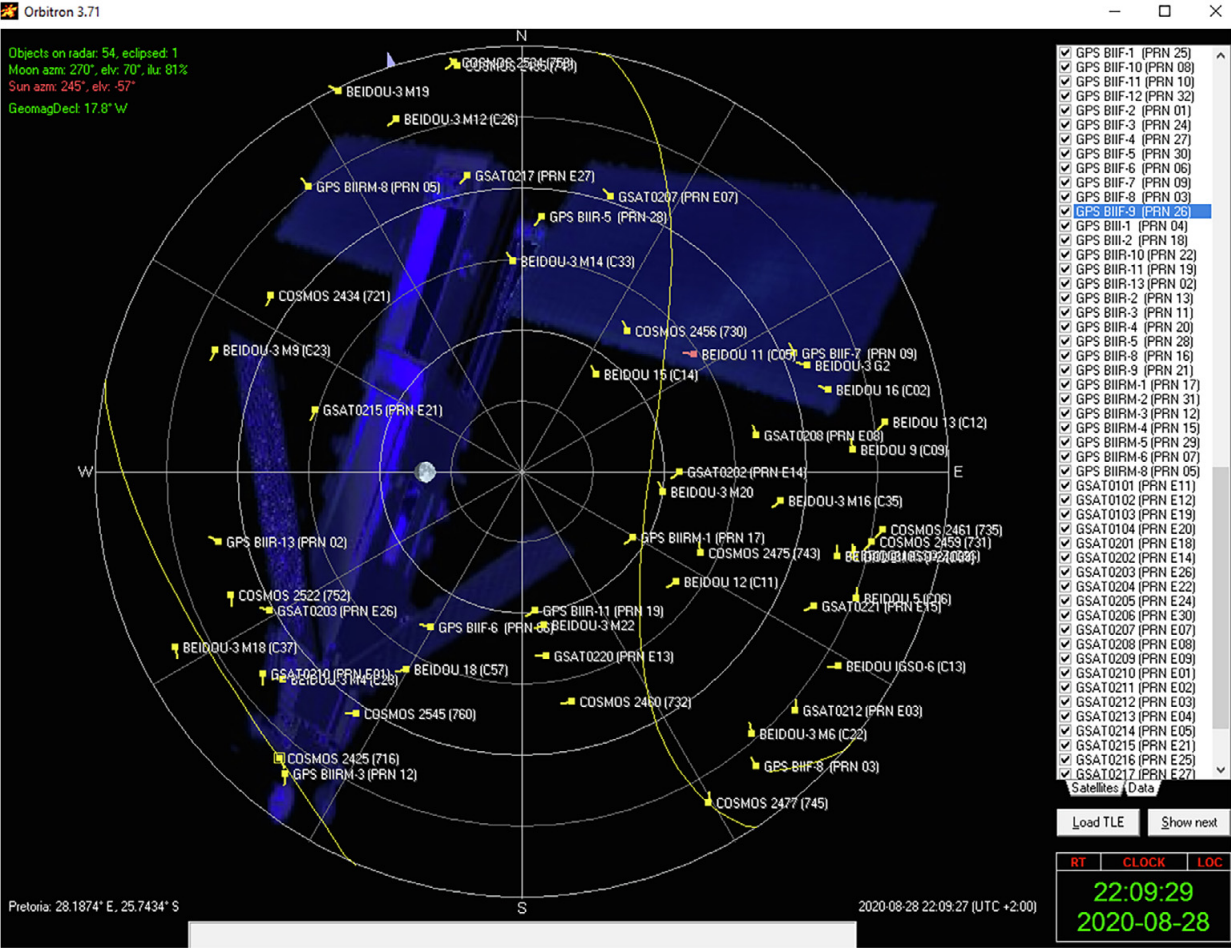
Predicted satellite observations visible from the measurement location (Pretoria, South Africa).

**Fig. 13:**
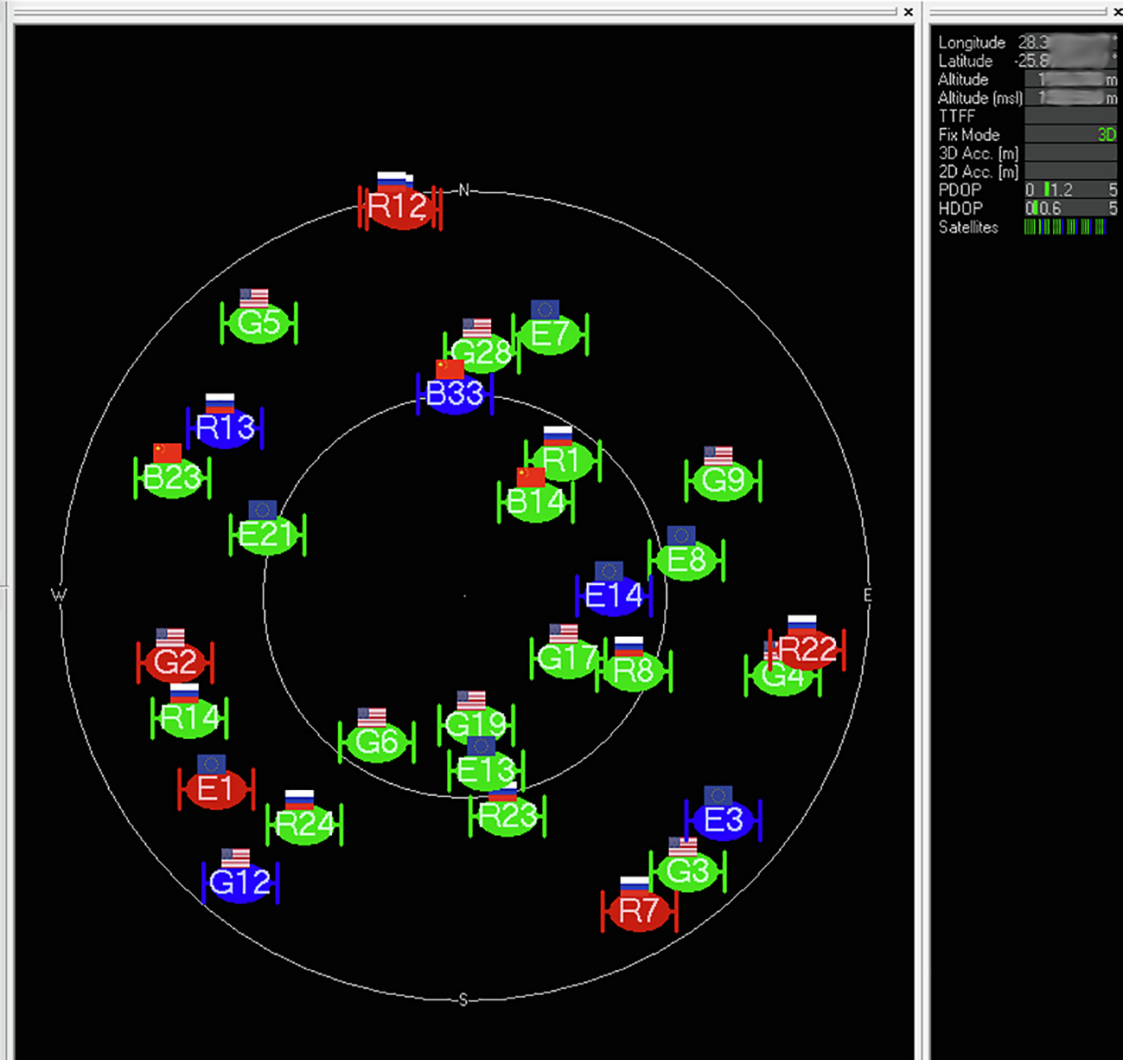
Satellite observations reported by the GNSS receiver from the measurement location (Pretoria, South Africa).

The BIOS (Basic Input/Output System) of the LattePanda was configured to always boot the system when power is applied, in the event that there is a prolonged failure, or the power is cycled remotely to restart the hardware. Additionally, the operating system was configured to never power down the computer, enter a sleep state or turn off the display. The installation files for SNIP [30], u-center [31], RTKLIB [32], Orbitron [47] and AnyDesk [48] were downloaded from the internet and installed on the computer. Orbitron serves as a convenient comparison between the known orbits of all operational GNSS satellites (Fig. 12) and observed satellites measured by the GNSS antenna (Fig. 13). Even though the latest revision of the software was released in 2005 (version 3.71), up-to-date NORAD (North American Aerospace Defense Command) two-line satellite telemetry data for all four the GNSS systems considered (GPS, GLONASS, Galileo and BeiDou) are available from CelesTrak [49].

### 6.2 Surveying of the antenna

**Fig. 14:**
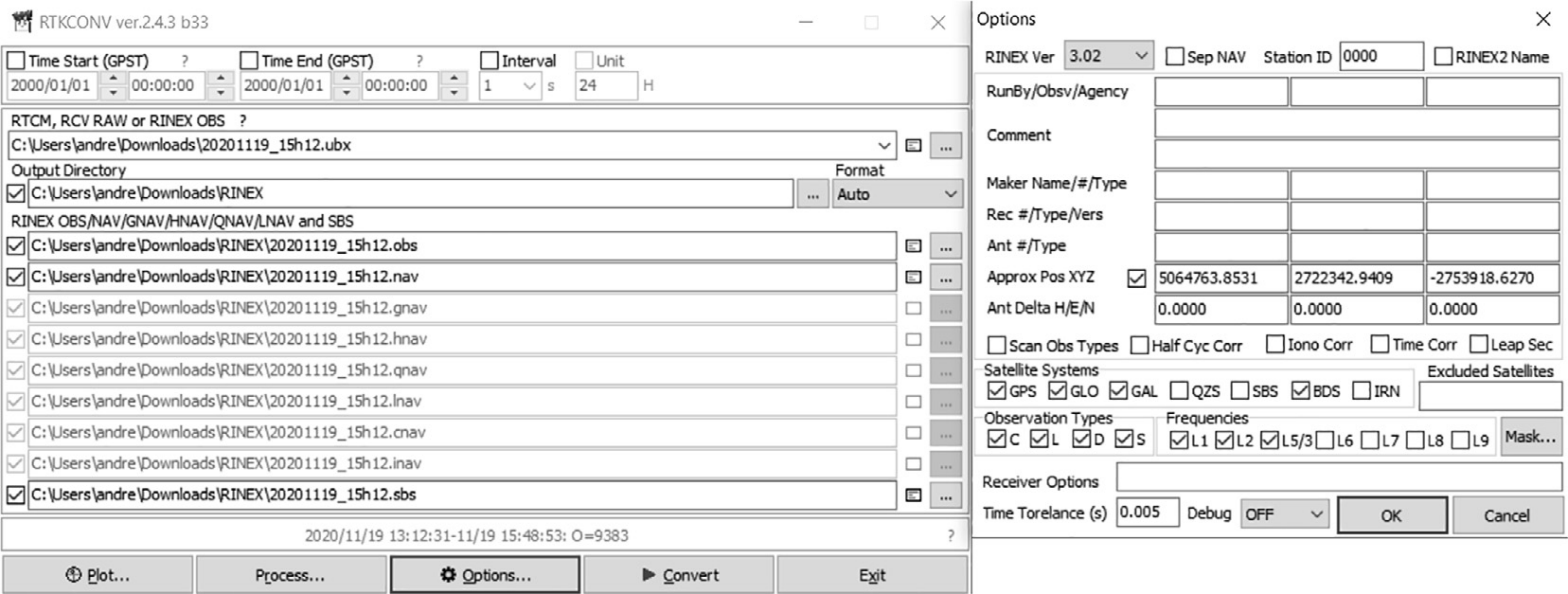
RTKLIB’s RTKCONV utility used to convert raw observation data into RINEX format.

**Fig. 15:**
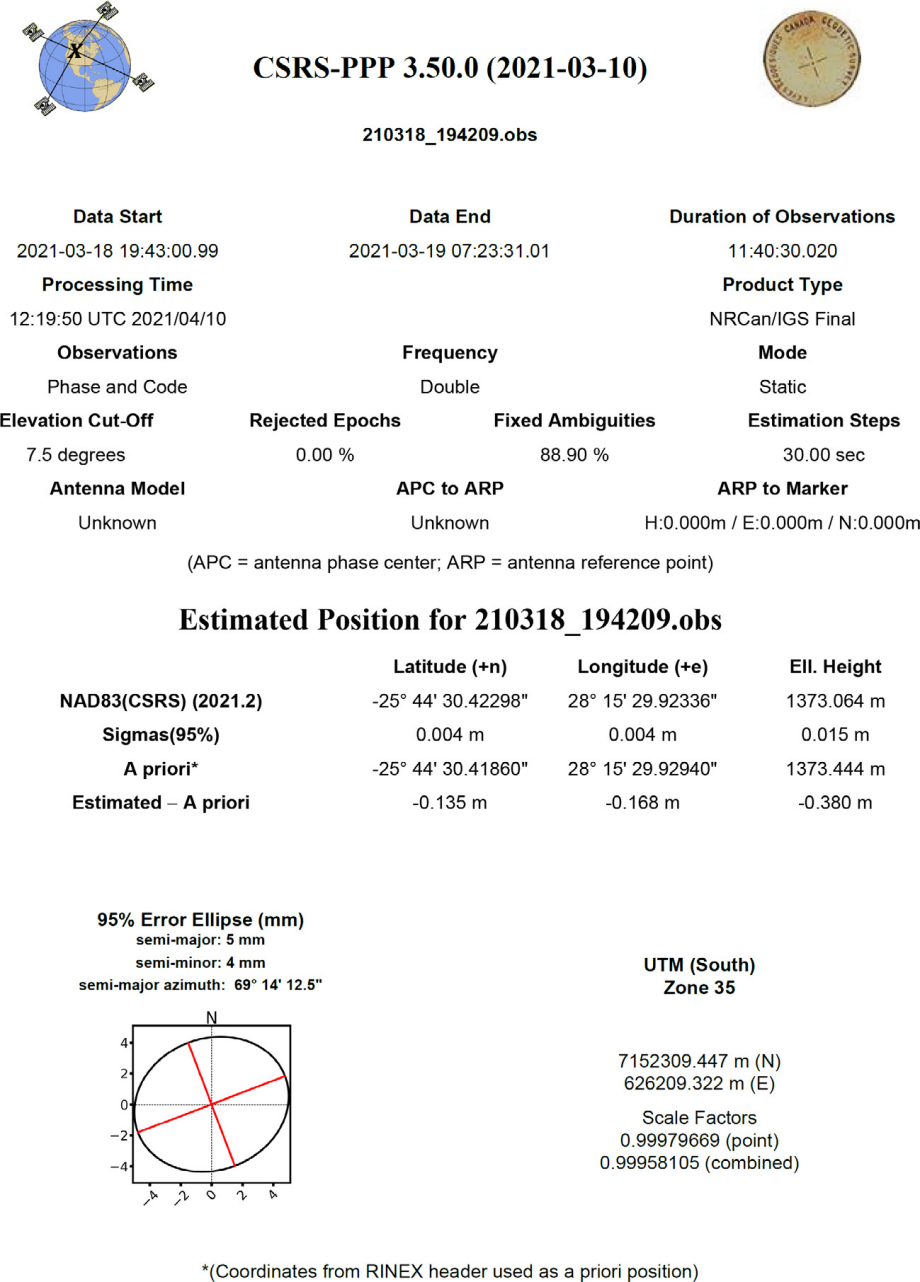
CRPS-PPP report generated from observation data 22 days after acquisition.

A video tutorial detailing the surveying process of the antenna is available from both the data repository (*Videos* folder) and YouTube [50] as part of a three-part series. Two survey modes are available:

• Averaging survey mode: After connecting to the GPS antenna within u-center, open the *Configure* window, and select the *TMODE3 (Time Mode 3)* option. To start the average survey-in process for the antenna, select the *1 – Survey-in* option from the *Mode* dropdown menu. The *Minimum Observation Time* and *Required Position Accuracy* must be configured as a minimum threshold criterion for the survey process to halt, with a value of 300 s and 1 m respectively, chosen as a default prior. Click on the *Send* button to send the survey settings to the GNSS receiver, initiating the survey process. Open the *Messages* window and navigate to *UBX – NAV (Navigation) – SVIN (Survey-In)*. Periodically click the *Poll* button to refresh the information. Once the survey-in process is completed, note down the *ECEF* (Earth-Centered Earth-Fixed) *X* ,*Y* and *Z* coordinates along with the *Mean 3D StdDev* (accuracy). Return to the *Configure* window and change *TMODE3*′s *Mode* to the *2 – Fixed Mode* option. After entering the three ECEF components and accuracy in the appropriate text boxes, click *Send* to reconfigure the GNSS receiver. The typical accuracy for this method is less than one meter.
• PPP: After connecting to the GPS antenna within u-center, open the *Configure* window and select the *MSG (Messages)* option. Select *02*–*15 RXM-RAWX* from the dropdown menu, check the *USB* checkbox and click *Send* to send the command. Click the *Record* button, select a location to save the raw measurement files (ubx file extension) and click *Yes* when presented with the window prompt to select the generation of receiver. Once the recording of data is completed (a minimum of 1 h is recommended, up to a maximum of 24 h), click the *Eject* button to terminate the process. Open RTKLIB’s *RTKCONV* utility and select the appropriate ubx input file (*RTCM, RCV RAW or RINEX OBS*) file, along with the desired output directory (*Output directory*) (Fig. 14). The *Options* menu requires selection of all four supported GNSS constellations (*Satellite Systems*), frequencies (*Frequencies*) and an approximated or coarse location. Click the *Convert* button on the main window to start the conversion process of the raw measurements into a RINEX observation file (obs file extension). The Canadian Spatial Reference System (CRPS) is an online PPP application [51] which is freely available to use for post-processing observation files. For maximum accuracy of the antenna’s position, up to two weeks are required for the convergence of historical satellite orbit ephemerides. Lower accuracy results can be generated within two hours of recording the observations. The RINEX observation file is uploaded to the server using the webpage, with the generated report file returned by email within a short period (Fig. 15). Similar to the first surveying method, the high accuracy PPP antenna coordinates can be configured for the GNSS receiver using the TMODE3 option in the *Configure* window. Provided a high-quality antenna, ground plane, largely noise-free environment and a sufficient number of measurements are used, an absolute accuracy of 7 mm can be realized using PPP. Note that the RTK rover’s reference datum is tied to the reference datum implemented by the reference station (RTK base station). The CRPS application allows the user to specify the reference datum, with the International Terrestrial Reference Frame (ITRF) recommended by default.

### 6.3 Configuration of the RTK base station and NTRIP server

A video tutorial detailing the configuration of the RTK base station and NTRIP server is available from both the data repository (*Videos* folder) and YouTube [52] as part of a three-part series. The configuration of the RTK base station and NTRIP server consists of two steps:

• Configure a sequence of *Messages* for the GNSS receiver after following the survey-in process as described earlier (the GNSS receiver’s *TMODE3* is configured for *2 – Fixed Mode* operation with a specified coordinate). Table 4 summarises the applicable *Messages* which require configuration for the GNSS receiver’s USB interface to operate in RTK mode. For the USB configuration, listed under *Ports* in the *Configure* window, both the *Protocol in* and *Protocol out* settings are selected as *0 + 1 + 5 - UBX + NMEA + RTCM3*. This final configuration can be permanently saved to the GNSS receiver within u-center (*Save Config*). Disconnect the GNSS receiver from within u-center to avail the Serial port for SNIP.

**Table 4:** RTK base station GNSS receiver message configuration for the USB interface.

USB interface enabled	USB interface disabled
F5-FD RTCM3.3 4072.1	02–15 FRMX-RAWX
F5-FE RTCM3.3 4072.0	02–13 RXM-SFRBX
F5-E6 RTCM3.3 1230	01–38 NAV-SVIN
F5-7F RTCM3.3 1127	01–03 NAV-STATUS
F5-61 RTCM3.3 1097	01–42 NAV-SLAS
F5-57 RTCM3.3 1087	01–43 NAV-SIG
F5-4D RTCM3.3 1077	01–32 NAV-SBAS
F5-05 RTCM3.3 1005	01–35 NAV-SAT
F0-00 NMEA GxGGA	01-3C NAV-RELPOSNED
F0-01 NMEA GxGLL	01–07 NAV-PVT
F0-02 NMEA GxGSA	01–02 NAV-POSLLH
F0-03 NMEA GxGSV	01–01 NAV-POSECEF
F0-04 GxRMC	01–14 NAV-HPPOSLLH
F0-05 GxVTG	01–13 NAV-HPPOSECEF
• SNIP is designed to ingest the serial data from the GNSS receiver, parse the data and push the corrections to the RTK2go [8] server over the internet. Registration of a uniquely-named mountpoint (and user credentials) on the RTK2go website [8] is required prior to configuring SNIP. Fig. 16 illustrates the typical configuration of both the *Serial Stream* (input) and *Push-Out Streams* (output); the geographic information and approximate location of the antenna is supplied, together with the selection of the four GNSS constellations. The *Mount Pt* name specified for the serial stream (Fig. 16, left) will appear as the only option in the drop-down list of the *Stream List* (Fig. 16, right); the *New MountPt Name* of the push out stream should be identical to the mountpoint name registered with RTK2go. The user credentials of the mountpoint registration should also be supplied (Fig. 16, right). Once the configuration is completed, right-click on the newly created *MountPt* in the *Serial Streams* tab and click the *Connect* option in the menu. If the configuration is working correctly, the *RTCM 3 Message Content Viewer* will reflect the statistics correctly (Fig. 17). Right click on the *Target* entry in the Pushed-Out Streams tab and click the *Connect* option to start the NTRIP server. If the connection to RTK2go is successful, the *Caster Status Report* webpage (Fig. 18) will return the status information correctly.

**Fig. 16:**
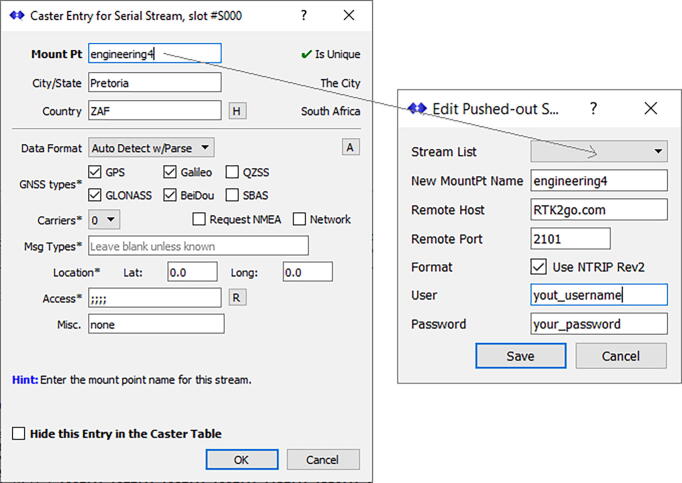
Example Serial Stream (left) and Pushed-Out Streams (right) configuration (SNIP).

**Fig. 17:**
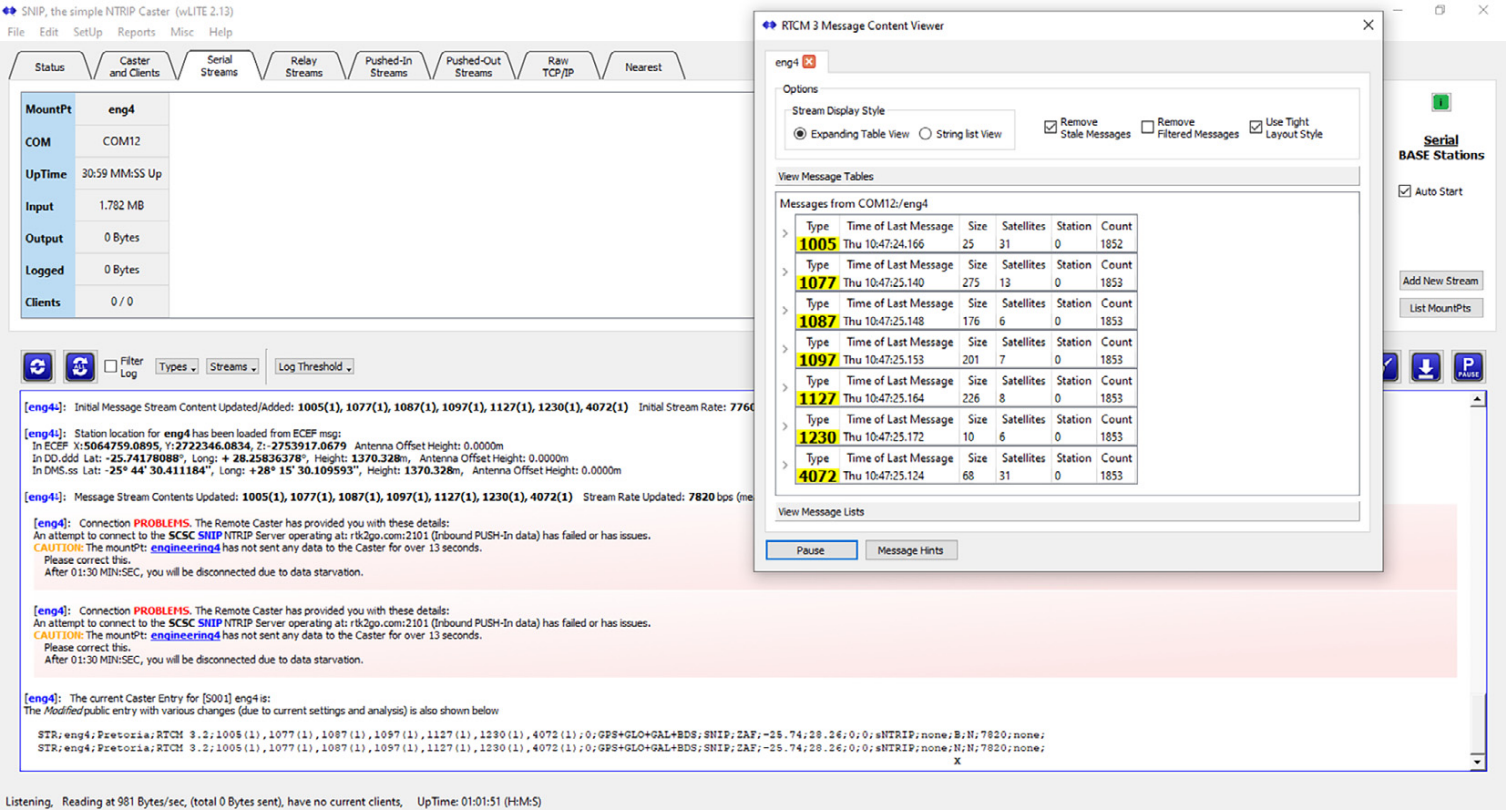
Example of correctly parsed RTCM 3 correction data (SNIP).

**Fig. 18:**
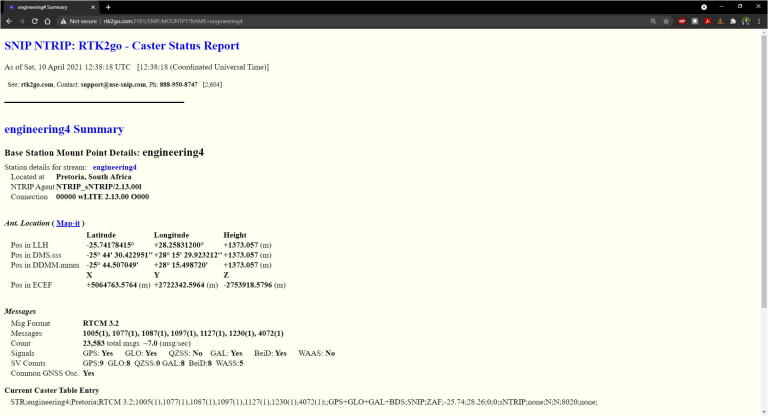
RTK2Go caster status report for an active engineering4 mountpoint.

### 6.4 Configuration of the RTK rover

A video tutorial detailing the configuration of the RTK base station and NTRIP server is available from both the data repository (*Videos* folder) and YouTube [53] as part of a three-part series. The update frequency of the GNSS receiver is configured to 8 Hz (*RATE* option in the *Configure* window), the maximum frequency for which all four GNSS constellation measurements are used in RTK mode to ensure the best accuracy. Table 5 summarises the applicable *Messages* which require configuration for the GNSS receiver’s USB and UART2 interfaces to operate in RTK mode. The Baudrate of the UART2 is configured to 115,200 to match that of the Bluetooth module. The *Protocol in* and *Protocol out* of the UART2 interface are configured as *0 + 1 + 5 - UBX + NMEA + RTCM3* and *0 + 1 - UBX + NMEA*, respectively. The *Protocol in* and *Protocol out* of the USB interface are configured as *0 + 1 + 5 - UBX + NMEA + RTCM3* and *1 – NMEA*, respectively.

**Table 5:** RTK rover GNSS receiver message configuration for the USB and UART2 interfaces.

USB and UART2 interface enabled	USB and UART2 interface disabled
F0-00 NMEA GxGGA	F5-FD RTCM3.3 4072.1
F0-01 NMEA GxGLL	F5-FE RTCM3.3 4072.0
F0-02 NMEA GxGSA	F5-E6 RTCM3.3 1230
F0-03 NMEA GxGSV	F5-7F RTCM3.3 1127
F0-04 GxRMC	F5-61 RTCM3.3 1097
F0-05 GxVTG	F5-57 RTCM3.3 1087
01–13 NAV-HPPOSECEF	F5-4D RTCM3.3 1077
01–02 NAV-POSLLH	F5-05 RTCM3.3 1005
	01-3C NAV-RELPOSNED
	01–03 NAV-STATUS
	01–07 NAV-PVT
	01–25 NAV-SAT
	01–43 NAV-SIG
	02–15 FRMX-RAWX
	02–13 RXM-SFRBX

### 6.5 NTRIP client software configuration

**Fig. 19:**
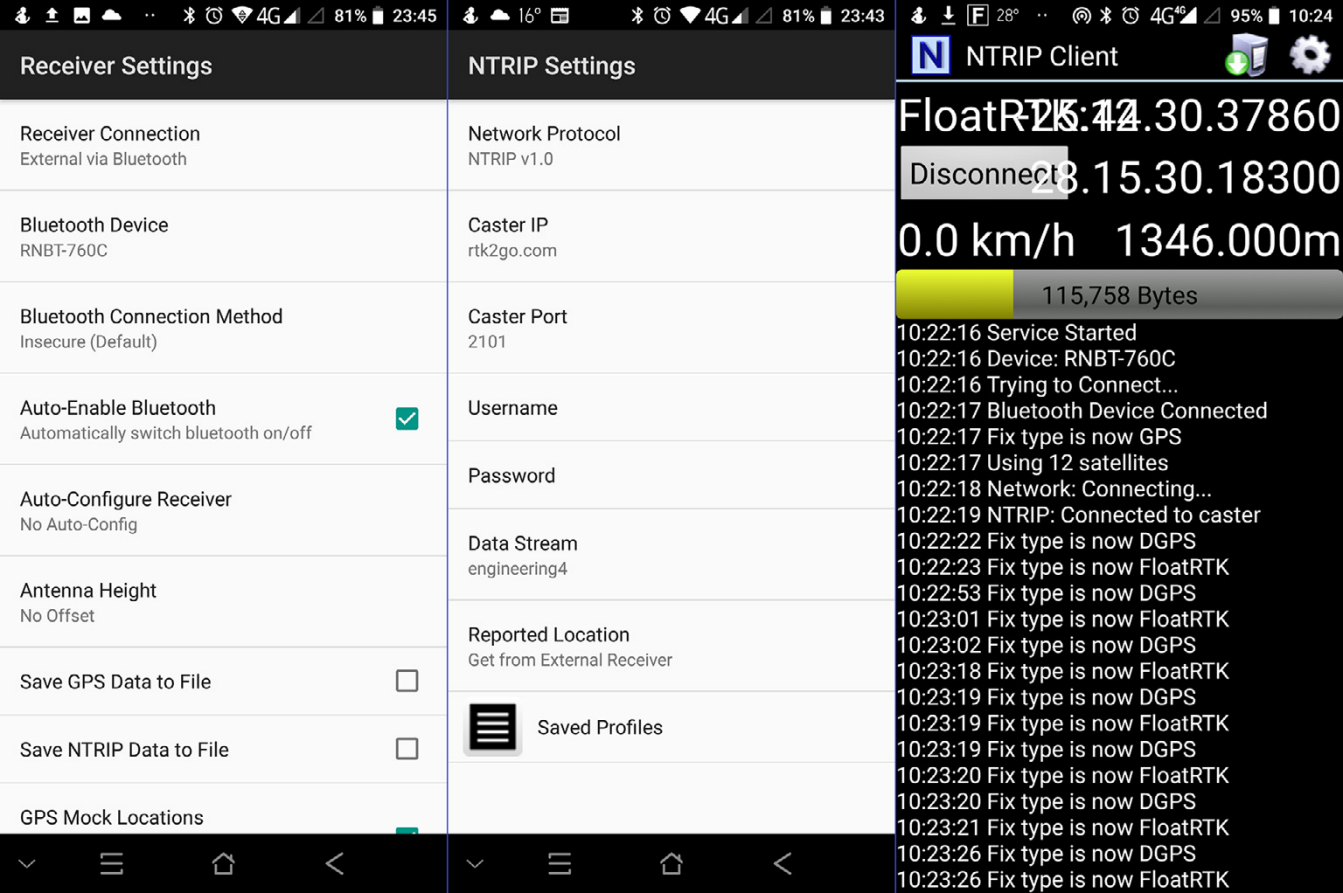
NTRIP Client Android application: Receiver Settings (left), NTRIP Settings (center) and NTRIP Client (right).

The *NTRIP Client* Android application available from Google Play [54] serves as the NTRIP client, sending corrections from the RTK2go server to the RTK rover’s GNSS receiver using the smartphone’s Bluetooth interface. Once the RTK rover’s Bluetooth module is paired with the Android device (Fig. 19, left) and the NTRIP server credentials specified (Fig. 19, center), the RTK corrections stream will be continuously sent to the rover (Fig. 19, center). The application serves as a useful visual indicator to confirm that an RTK fix is achieved, with optional settings to display navigation information (of the RTK rover).

### 6.6 RTK rover operation modes

The Arduino firmware was designed to operate in two possible configurations:

• Single-shot: data is only recorded when the external trigger is engaged. If the external trigger is engaged, data will be recorded at the maximum possible frequency (approximately 2.5 Hz) until the trigger is released. This mode is selected when the external trigger is not engaged during the boot sequence of the microcontroller. This mode is preferred for handheld measurements or surveying applications (refer to the monument digitisation example as part of the validation process).
• Continuous: data is continuously recorded, irrespective of whether the trigger is engaged or not. Data will be recorded at the maximum possible frequency (approximately 2.5 Hz) until the power is switched off. This mode is selected when the external trigger is engaged during the boot sequence of the microcontroller. This mode is preferred for vehicle measurements where continuous data is required, or where the operator cannot operate the controls (refer to the RTK coverage mapping example as part of the validation process).

The primary constraint in achieving a higher logging frequency is the OLED display. Removing the OLED display entirely in favour of simplified LED indicators increased the logging frequency to approximately 4 Hz. The following variables are stored in the *GPS.csv* file on the SD card, irrespective of the operating mode used:

• Unixtime (Integer) [s];
• Record counter (Integer);
• DateTime to the nearest millisecond (String, YYYY/MM/DD HH:MM:SS.mmm);
• Latitude (Float, 8 decimals);
• Longitude (Float, 8 decimals);
• Hight above the ellipsoid [m] (Float, 3 decimals);
• Height above the mean sea level [m] (Float, 3 decimals);
• Satellites in view (Integer);
• Horizontal accuracy [m] (Float, 4 decimals);
• Vertical accuracy [m] (Float, 4 decimals);
• Ground speed [mm/s] (Integer);
• Heading [deg] (Integer);
• IMU roll [deg] (Float, 1 decimal);
• IMU pitch [deg] (Float, 1 decimal);
• IMU yaw [deg] (Float, 1 decimal);
• IMU quaternion (i-component) (Float, 5 decimals);
• IMU quaternion (j-component) (Float, 5 decimals);
• IMU quaternion (k-component) (Float, 5 decimals), and
• IMU quaternion (r-component) (Float, 5 decimals).

## 7 Validation and Characterization

The demonstration of the RTK performance was evaluated for three scenarios: RTK coverage over a large geographic area in an urban environment, chord measurements of road infrastructure and small-scale feature measurements of a monument as a potential substitute for traditional LiDAR and photogrammetry applications. A fourth configuration compares measurements obtained from the RTK rover with that of a digital level. Note that the final information obtained from the receiver is limited to the geolocation data with specifics such as the individual satellite counts not considered.

### 7.1 Accuracy evaluation

Recently, the u-blox ZED-F9P performance was thoroughly evaluated by the University of Ljubljana in Slovenia [55]. The aim of this specific study was to evaluate the noise of low-cost GNSS receivers, compare the positioning quality from different low-cost antennas and to analyse the positioning differences between low-cost and geodetic receivers. The zero-baseline test for the u-blox ZED-F9P indicated low noise within the submillimetre range. The influence of antenna selection (Tallysman TW3882 and survey antenna) demonstrated the superior horizontal positioning accuracy of the Tallysman antenna (0.1 mm) compared to the survey grade antenna (1.0 mm). The differences for the ellipsoid height were measured as 0.6 mm and 0.3 mm for the Tallysman and survey antenna, respectively. For the same low-cost antenna, geodetic instruments yielded improved performance compared to the ZED-F9P receiver. The performance of the low-cost receivers and antennas provide sufficient for various geodetic applications. No information pertaining to the modelling of the atmospheric delays or integer ambiguity resolving methods is provided by either the authors of this study nor the manufacturer.

**Fig. 20:**
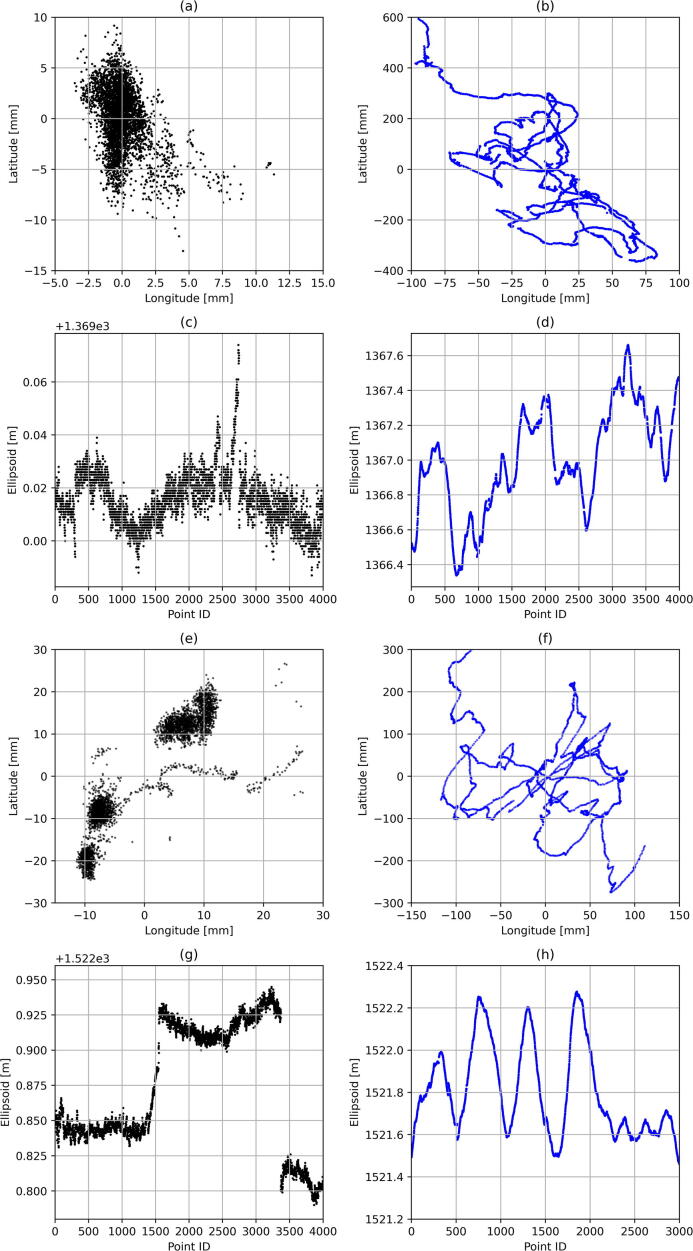
Accuracy comparison for measurements with (left column) and without RTK corrections (right column) at Engineering 4.0 (a-d) and a location 11 km away from the reference antenna (e-h).

Two sets of static measurements were acquired, the first 12 m away from the reference antenna installed at the Data House (3 989 samples), with a second at a distance of 11.416 km (4 748 samples). The prism pole with the RTK rover (Fig. 3, left) was securely fixed to a tripod to allow clear line-of-sight with the satellite constellations. The largest geometric distance between any two measurement points defines the accuracy as it can be assumed that the antenna position resides within the convex hull enclosing the set of measurement vectors. The best-case scenario – referring to the measurements recorded directly adjacent to the Data House – yields a 22.8 mm accuracy for the horizontal plane (Fig. 20a) and 87.0 mm for the elevation (Fig. 20c). With RTK corrections removed, the accuracy degrades to 972 mm for the horizontal plane (Fig. 20b) and 1322 mm for the elevation (Fig. 20d). For the measurements located some 11.4 km away from the reference antenna, a 60.9 mm and 155 mm accuracy are achieved for the horizontal plane (Fig. 20e) and elevation (Fig. 20g), respectively. With the RTK correction disabled, the horizontal accuracy reduces to 596 mm for the horizontal plane (Fig. 20f) and 961 mm for the elevation (Fig. 20h).

### 7.2 RTK coverage

**Fig. 21:**
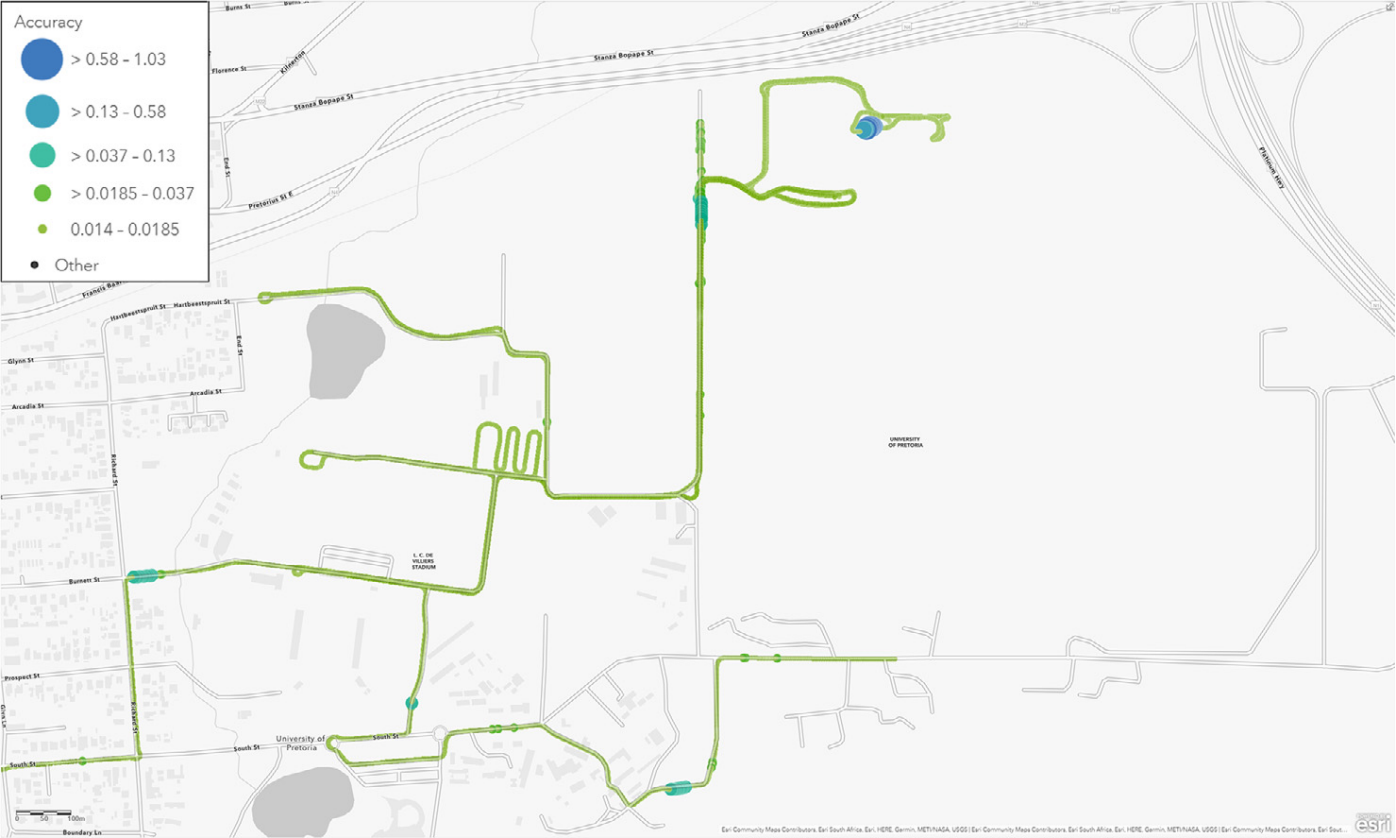
Map of the Hillcrest campus illustrating the RTK horizontal accuracy.

**Fig. 22:**
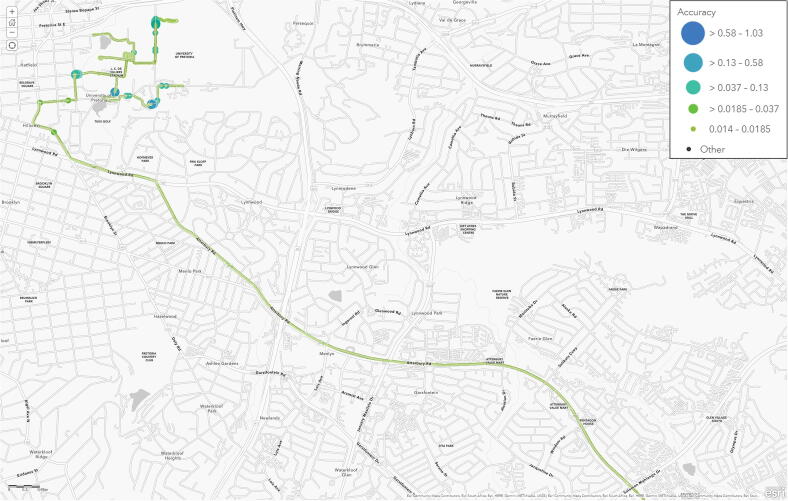
Map of Pretoria East illustrating the RTK horizontal accuracy.

The RTK coverage was evaluated using two trips undertaken with a sedan motor vehicle. The Tallysman TW1829 antenna was substituted in favour of the u-blox ANN-MB-00 [56] magnetic mount patch antenna, which was installed on the roof of the vehicle along the middle of the wheelbase. Save for the occasional tree-lined road, the horizontal accuracy for the RTK measurements remained nearly constant (14 mm), whether in close proximity on the Hillcrest campus (Fig. 21, 5 431 samples) or up to 9 km away from the RTK base station (Fig. 22, 3 369 samples). The 14 mm RTK performance was validated at a maximum distance of 15 km (near Highveld, Centurion).

### 7.3 Road geometry

Instead of using a motor vehicle to measure the geometry of road infrastructure with the RTK rover, a golfcart was selected owing to the smaller wheelbase measuring 1.7 m, increasing the sensitivity of the chord measurements. The u-blox ANN-MB-00 was glued to the roof, measuring 1.7 m above the ground. A speed arrestor (aligned in an East-West direction) was selected as the measurement target, with the crest (3.3 m) wider than the wheelbase of the golfcart.

**Fig. 23:**
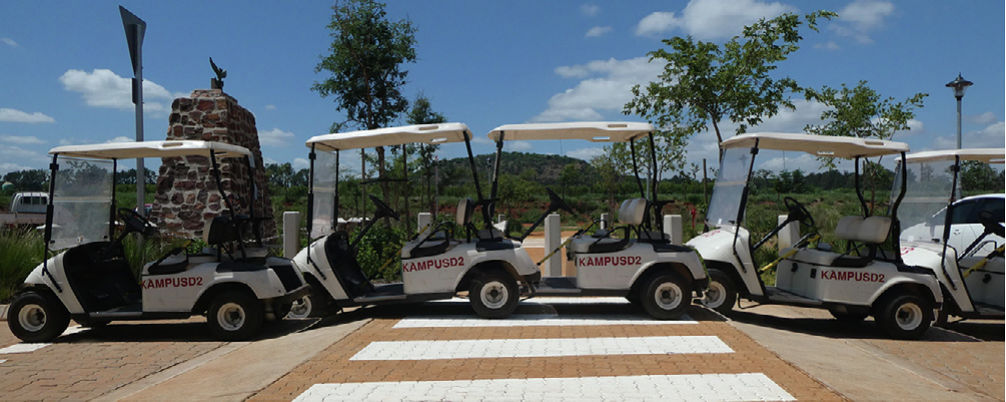
Composite of the golfcart chord measurements.

**Fig. 24:**
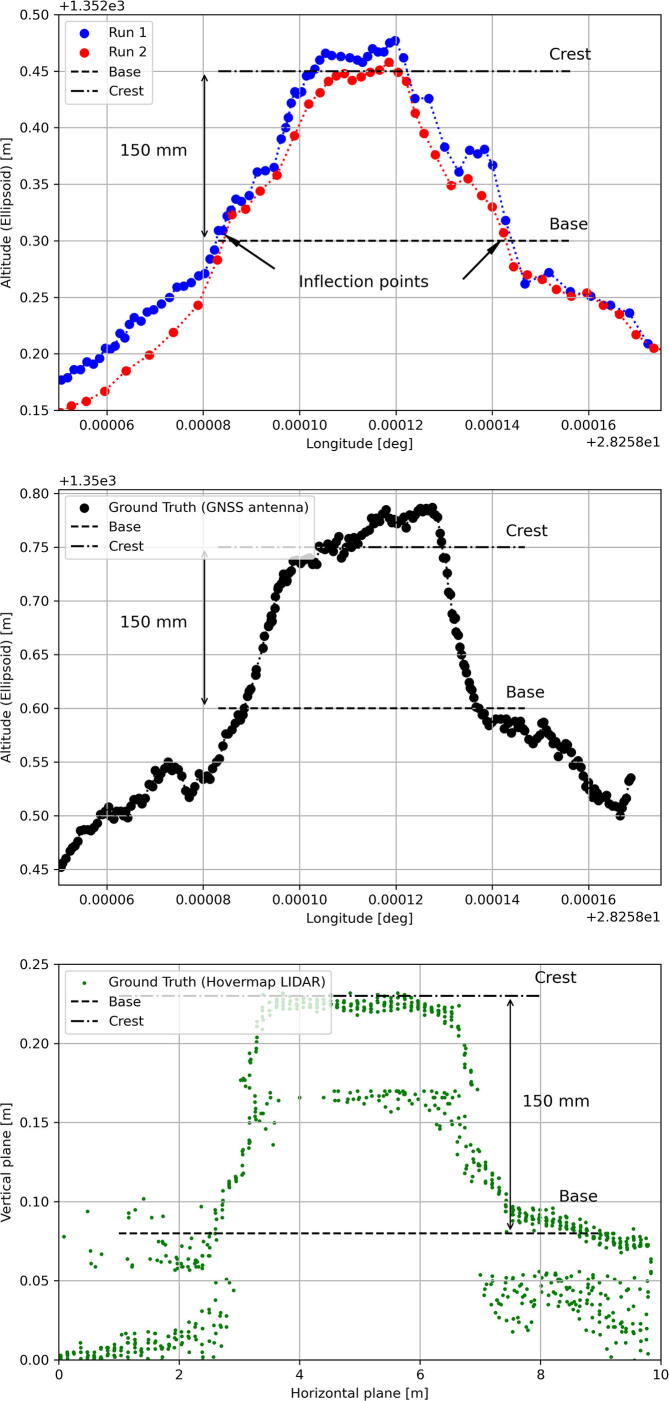
Geometry measurements of the speed arrestor using the GNSS antenna mounted to the roof of the golfcart (top), dragging the antenna over the ground (center) and Hovermap LiDAR (bottom).

**Fig. 25:**
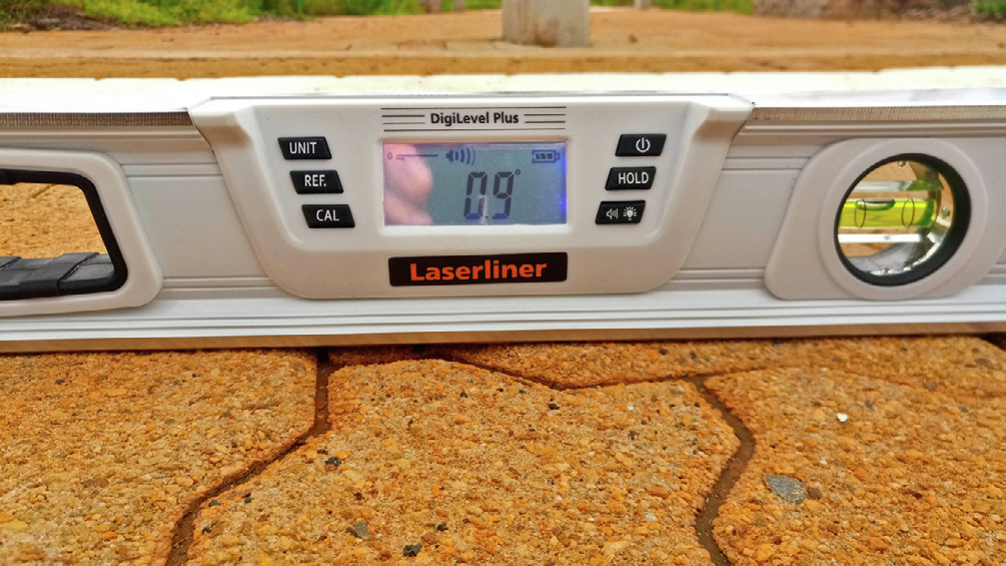
Digital spirit level positioned along the center of the speed arrestor.

Fig. 23 illustrates a composite image of the path travelled by the golfcart. Fig. 24 (top) illustrates the measured vertical alignment of the speed arrestor as a function of the longitude, with two runs conducted in opposing directions (345 points). The inflection points of the chord measurements denote the position where the golfcart is in contact with the base of the speed arrestor. The approximated height of the speed arrestor as measured by the RTK rover (150 mm) is nearly identical to the 145 mm measurement using a digital level and tape measure. For comparison, the GNSS antenna was slowly dragged over the speed arrestor (Fig. 24, center), along the same path followed by the golfcart, serving as a well-defined ground truth (225 points). For convenience, this method of measuring is referred to as RTK antenna surface mapping (RTK-ASM). An elevation difference of approximately 50 mm is observed over the width of the crest as measured by the GNSS receiver (Fig. 24, center). This difference was validated by positioning a calibrated, digital spirit level at the center point of the speed arrestor (Fig. 25). If the tangent of the level measurement (0.9°) is multiplied by the crest width of the speed arrestor (3 300 mm), the resulting 51.8 mm offset matches the RTK-ASM observations nearly perfectly.

High accuracy LiDAR measurements were recorded using the Emesent Hovermap [57] (without a reference datum), serving as an independent external measurement reference (Fig. 24, bottom). The LiDAR puck of the Hovermap scans 300 000 points per second over a 360°×360° field of view with an accuracy and repeatability of ± 30 mm and ± 10 mm, respectively. Some ambiguity is noticeable in the resulting point cloud (1 057 points), most likely attributed to the reflective white paint, although the same profile is clearly visible compared to the two RTK rover measurements.

### 7.4 Monument digitisation

**Fig. 26:**
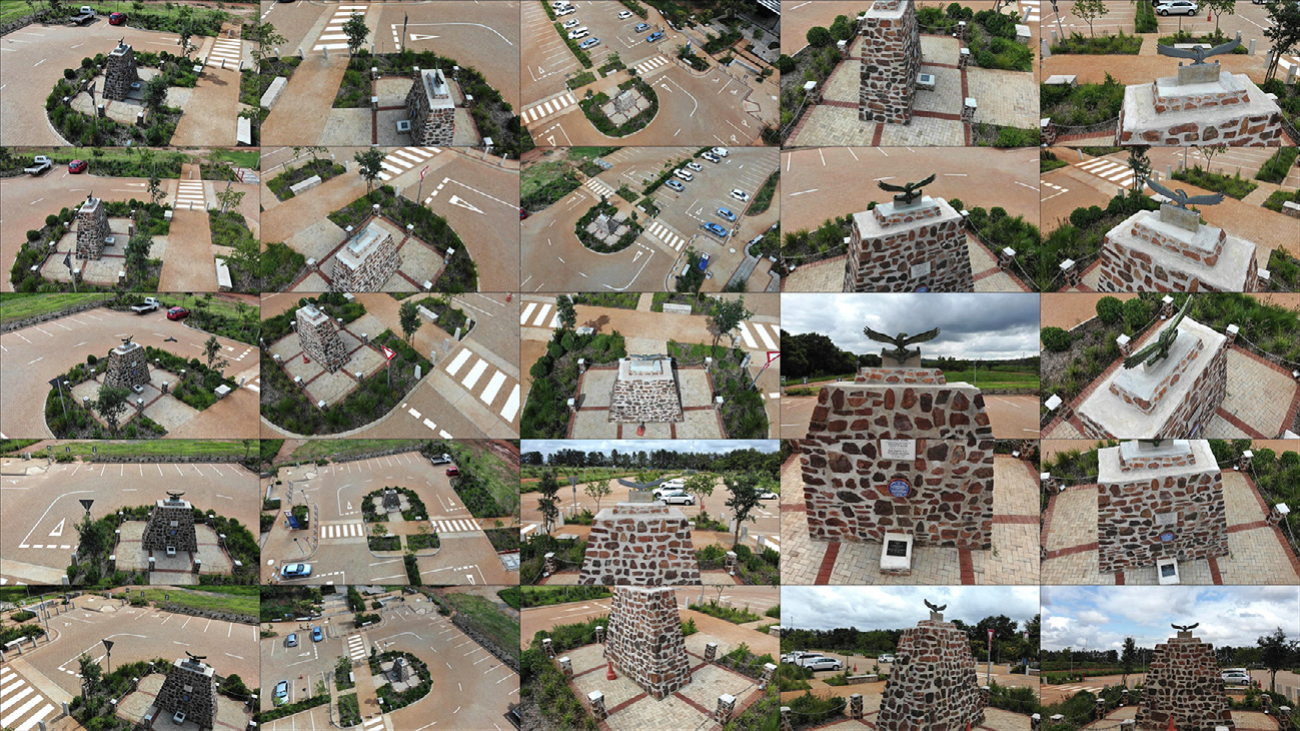
Sample of the monument photographs collected for photogrammetric reconstruction.
• Emesent Hovermap LiDAR installed on the front of a MultiOne utility vehicle (Fig. 27), and

The Pierre Van Ryneveld monument was unveiled at its third location on 14 March 2020, shortly after the completion of the Engineering 4.0 facility on 28 February 2020. The monument commemorates the first *trans*-Africa flight from London to South Africa undertaken by Sir Pierre Van Ryneveld and Sir Quintin Brand in 1920 [58]. The monument, pyramidal in shape and constructed from rocks and concrete mortar, stands approximately 3.5 m × 1.6 m × 3 m in size. This also serves as a permanent fixture ideal for digital preservation. The model was measured using three techniques:

• Photogrammetry using a DJI Mavic Enterprise UAV (Autodesk ReCap, 100 photographs) (Fig. 26);

**Fig. 27:**
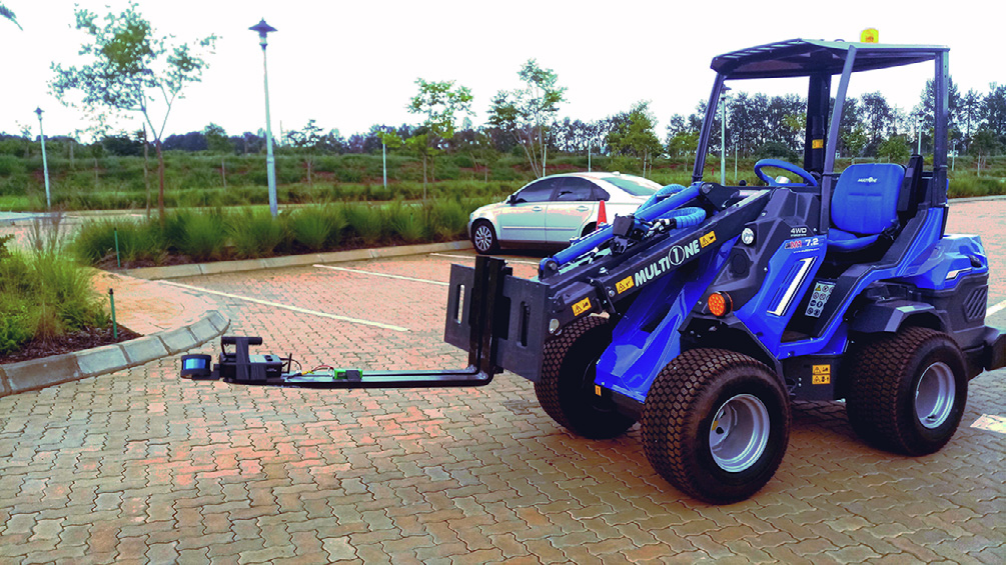
Hovermap LiDAR fixed to the front of the utility vehicle.
• RTK-ASM measurements using the RTK rover (Fig. 28).

**Fig. 28:**
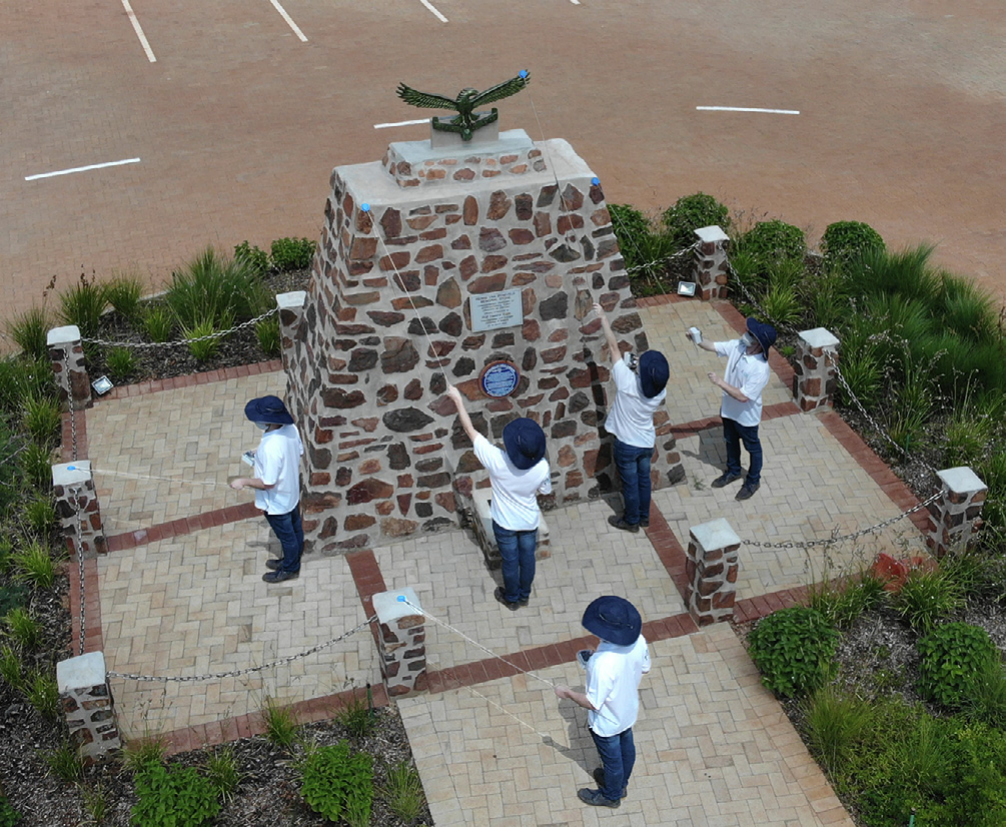
Composite image of the RTK-ASM measurements (pole-mounted GNSS antenna).

**Fig. 29:**
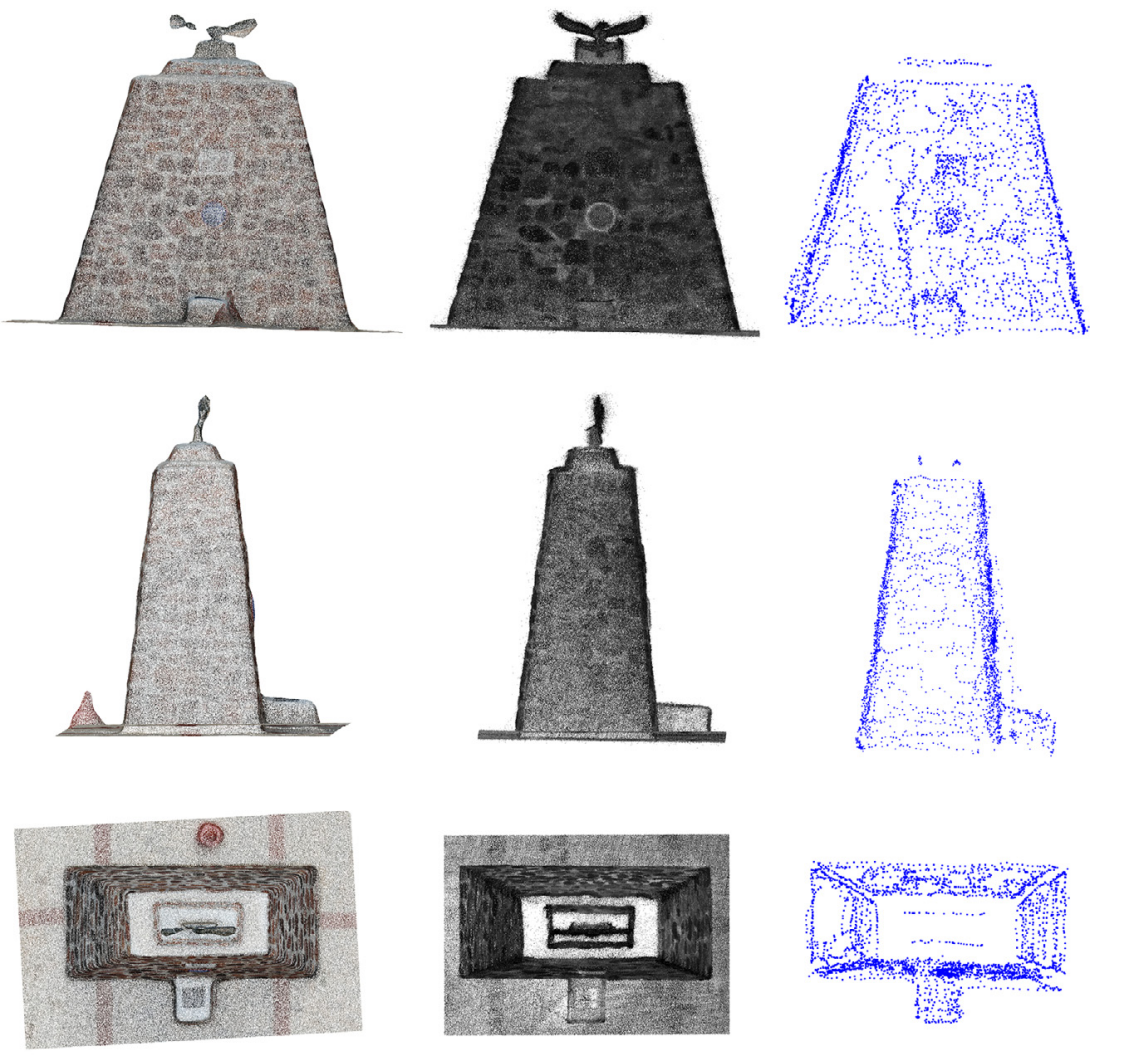
Pierre Van Ryneveld reconstruction from aerial photogrammetry (left column), Hovermap LiDAR (center column) and RTK-ASM (right column) as viewed from the front (first row), side (second row) and top (third row).

**Fig. 30:**
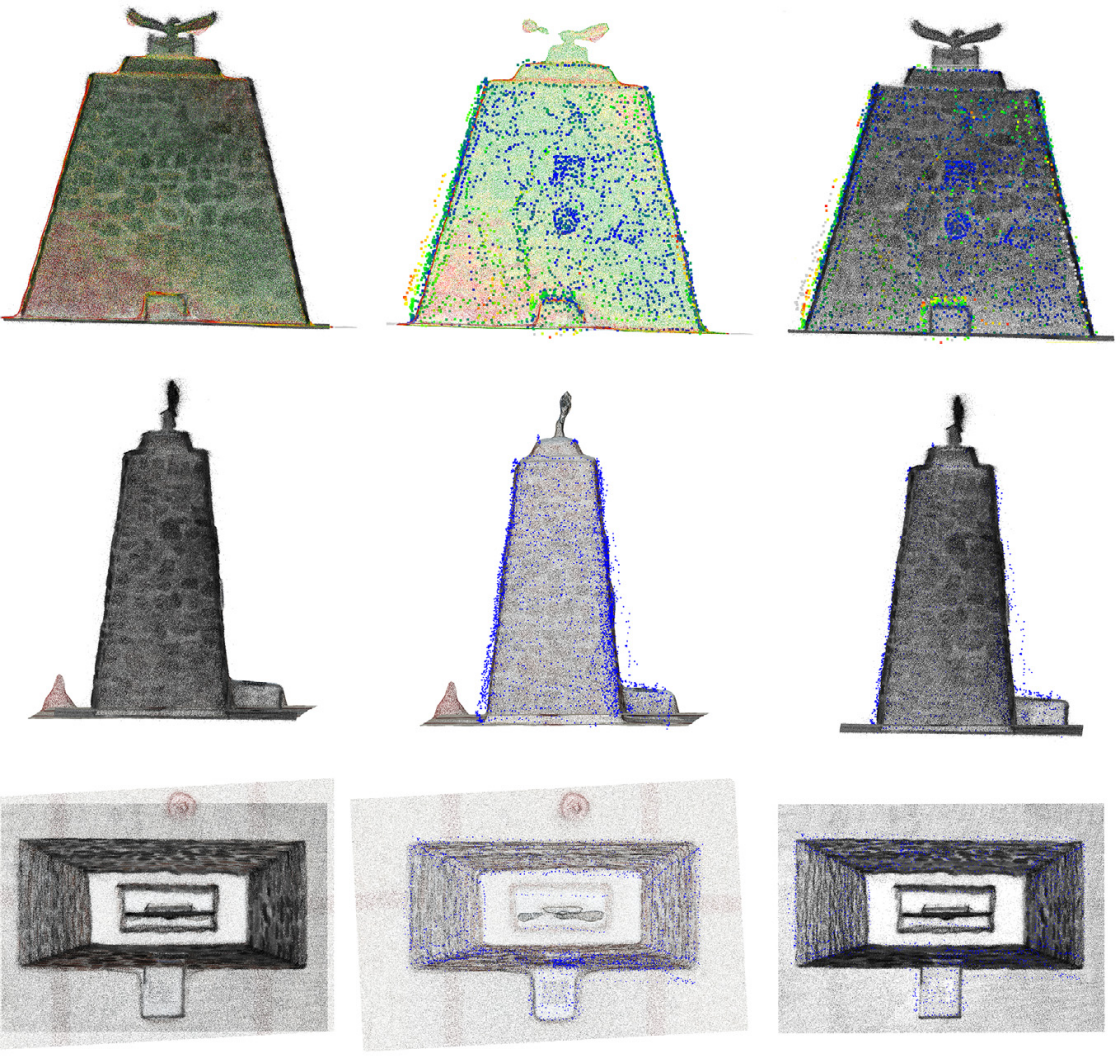
Pierre Van Ryneveld cloud-to-cloud comparisons for LiDAR-photogrammetry (left column), photogrammetry-RTK-ASM (center column) and LiDAR-RTK-ASM (right column) as viewed from the front (first row), side (second row) and top (third row).

The three measurement techniques each proved unique in their processing pipeline and overall quality of reconstruction/measurement. The photogrammetric method benefits from AutoDesk’s cloud processing facility but limits the number of photographs to 100. High frequency details were absent from the reconstructed mesh (19 751 points) despite the close proximity between the UAV and the monument for a number of photographs (Fig. 26). The Hovermap LiDAR proved to be the fastest method with a detailed scan completed within 5 min, compared to the 15–20 min required for the UAV and 2 h for RTK-ASM. Hovermap’s post-processing software produced the highest point cloud density by a significant margin (3.329 million points) with detailed coverage of the surrounding area (range up to 100 m, cropped for accurate comparisons). The RTK-ASM method did provide increased flexibility to scan areas of interest with higher detail whilst retaining the ability to resume or pause the scan at any point in time, irrespective of illumination or weather conditions which can hamper the other two methods. RTK-ASM does however produce a low-density point cloud (3 145 points), combined with the small offset between the surface geometry and antenna’s phase center reducing the overall accuracy, more so for non-planar geometry. The three point clouds (randomized points were sampled from the photogrammetry mesh) were aligned and rescaled as accurately as possible using CloudCompare [59], with the RTK-ASM model serving as a ground truth reference for the correct scale (Fig. 29). CloudCompare’s cloud-to-cloud distance metric was implemented as a quantitative method to evaluate the difference between the point cloud pairs, namely LiDAR-photogrammetry, photogrammetry-RTK-ASM and LiDAR-RTK-ASM (Fig. 30). For this application, the Hovermap LiDAR is chosen as the ground truth reference instead of the photogrammetry reconstruction, due to the improved representation of the monument’s geometry. When comparing the cloud-to-cloud distance between the RTK-ASM and LiDAR point cloud (Table 6), the mean difference of only 10 mm highlights the repeatability and accuracy attained with the RTK rover when referenced to a much more sophisticated and expensive optical sensor.

**Table 6:** Cloud-to-cloud distance statistics for the three comparison pairs

Cloud-to-cloud distance	50th percentile	75th percentile	95th percentile
LiDAR-photogrammetry	17 mm	28 mm	48 mm
Photogrammetry-RTK-ASM	12 mm	55 mm	98 mm
LiDAR-RTK-ASM	10 mm	24 mm	67 mm

### 7.5 Levelling

The Experimental Test Track [60] located on the Hillcrest campus of the University of Pretoria serves as the location to demonstrate the quantitative performance of the RTK functionality compared to precision optical instrumentation. The 30 m section of PY Slab Track investigated consists of 48 kg/m, 57 kg/m and 60 kg/m rail segments each measuring 10 m in length. The vertical geometry, defined as the highest point perpendicular to the rail, is used to calculate the variability of the geometry and resulting riding quality.

**Fig. 31:**
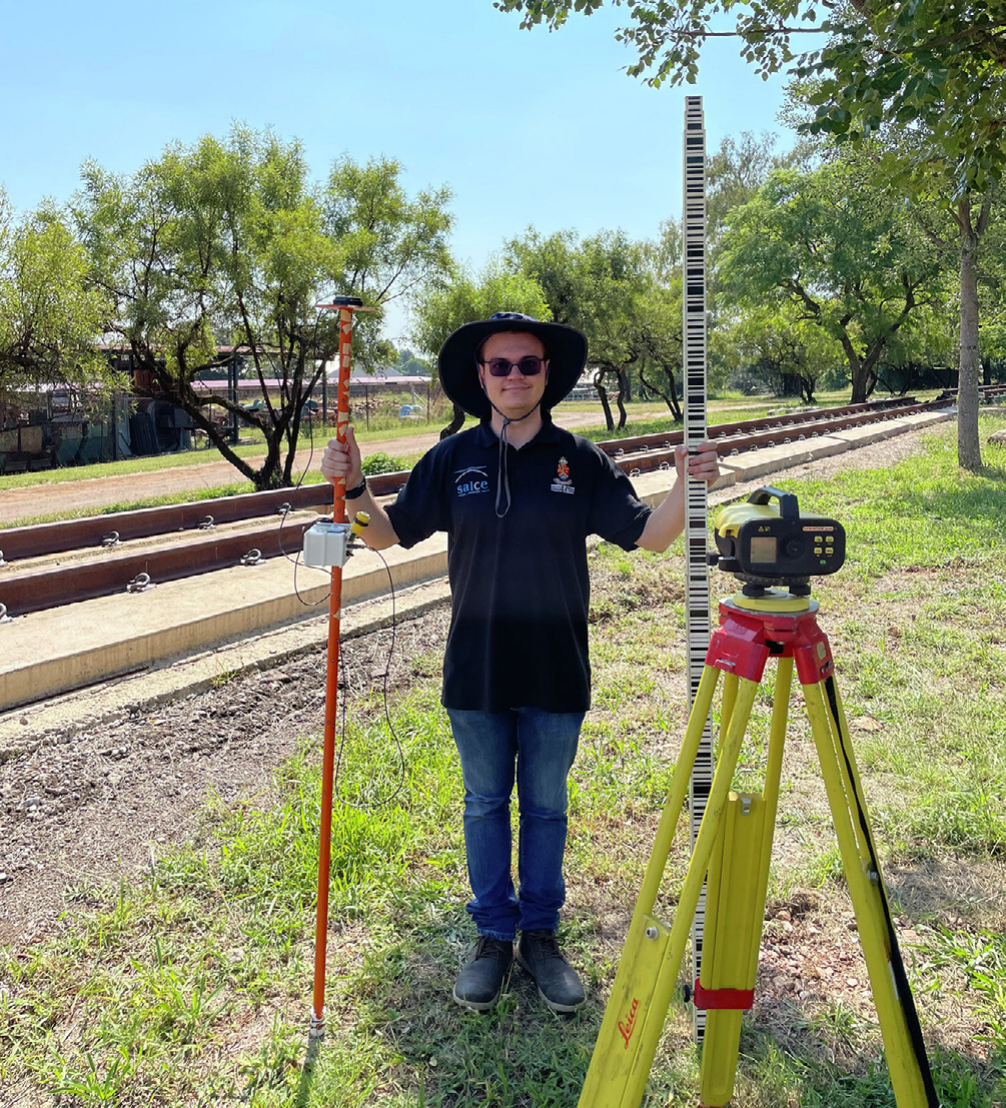
RTK rover (left) and digital level (right) instruments used to survey the PY slab track (background).

**Fig. 32:**
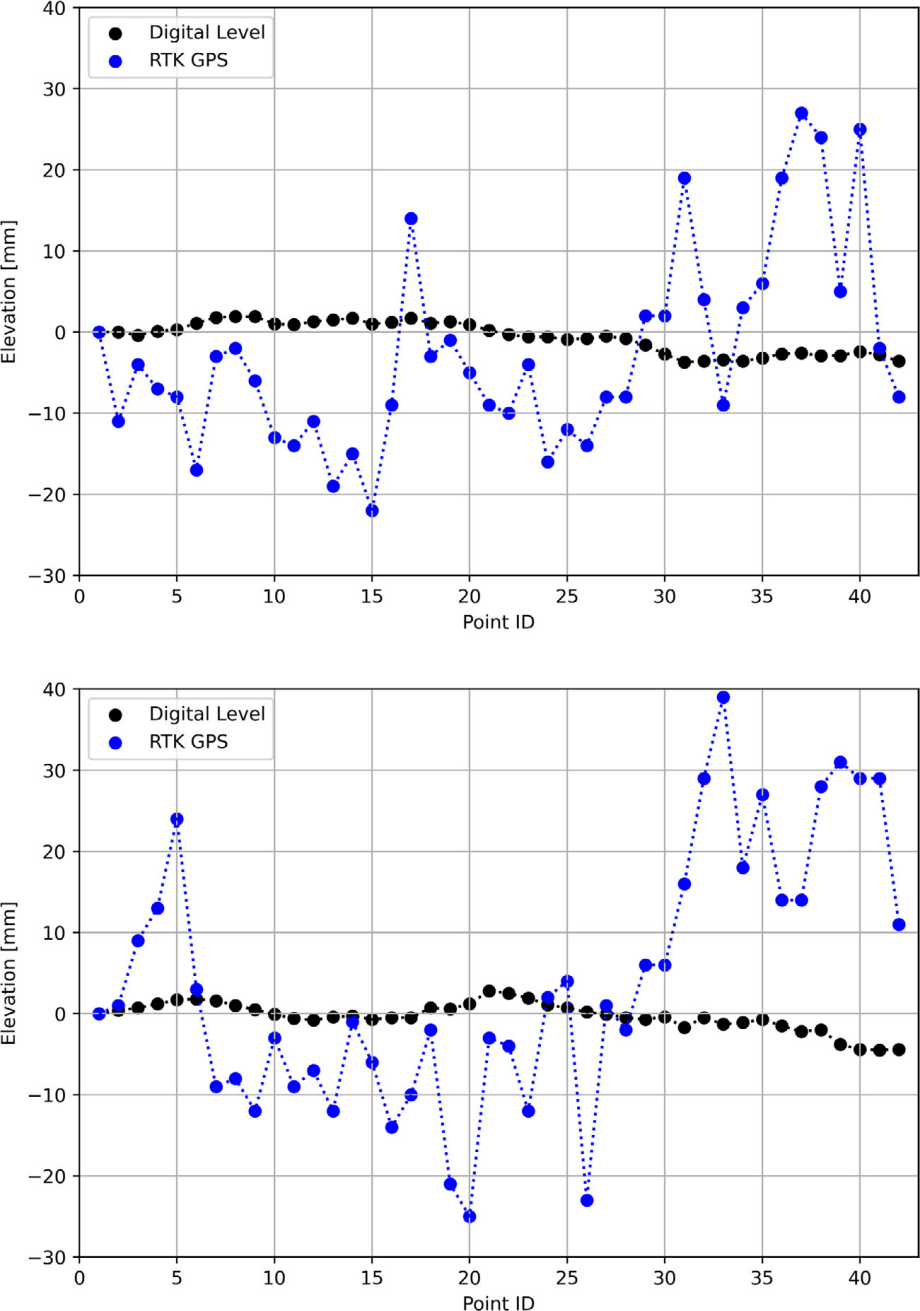
Comparison between the digital level and RTK GPS measurements for the left (top) and right (bottom) rail.

The Leica Sprinter 250 M digital level (Fig. 31, left), with a stated accuracy of 0.7 mm, and RTK rover (Fig. 31, right), was used to measure 42 points along each of the rail profiles (Fig. 31, background), spaced equidistantly (710 mm center-to-center) by the e-clip fasters, for a total of 84 measurement points. The u-blox ANN-MB-00 magnetic mount patch antenna was utilised for the GPS RTK measurements. The sparse population of trees did not exhibit a detrimental influence over the measurements. Fig. 32 illustrates the comparison between the two measurement techniques for both the left-side (Fig. 32, top) and right-side (Fig. 32, bottom) rail profiles (left-side refers to the rail profile located closest to North). Vertical accuracy, defined as the maximum difference between the true (digital level) and observed (RTK GPS) geometry, is calculated to be 40.3 mm, with the expected measurement error (50th percentile) as 9.35 mm. Horizontal accuracy, defined as the maximum difference between the mean center-to-center e-clip spacing and observed (RTK GPS) geometry), is calculated to be 49.7 mm, with the expected measurement error (50th percentile) as 20.6 mm. Considering some degree of noise is introduced owing to the manual measurements, alongside the kinematic antenna, the ensuing accuracies presented are representative of the values obtained from the static accuracy verification process.

Based on the practical experience gained from development process and field performance together with the four field experiments, the following capabilities and limitations of the RTK geolocation service is summarized:

• Reliable RTK performance was achieved with a horizontal accuracy of 14 mm, irrespective of the distance from the RTK base station;
• Comparatively low-cost GNSS receivers and small form factor computers provide a viable alternative for commercial hardware solutions to develop a highly customizable RTK-grade geolocation service;
• The presented implementation allows for the hardware to be relocated to a remote location for RTK measurements. For example, measuring the structural response of wind turbines either on– or offshore where rapid survey-in methods can be utilised;
• The mobility presented by the RTK rover presents unique opportunities to enhance research projects, ranging from road and rail geometry measurements, long-term ground heave or settlement, digitisation of historical monuments, agricultural applications and sports engineering;
• RTK-ASM serves as a natural progression in the field of measurement techniques, complementing existing LiDAR and photogrammetric reconstruction methods with comparable if not better accuracy;
• RTK measurements provide both centimetre relative and absolute accuracy to a specified reference datum;
• PPP applications are freely available online to survey stationary GNSS antennae with mm-grade accuracy;
• The u-blox ZED-F9P GNSS receivers do support a moving RTK base station configuration; this option has not been explored, and
• Only the total number of satellites could be obtained from the rover’s receiver, not that of the individual constellations.
• A suitable, permanent location with electrical power and internet connectivity is required for the RTK base station hardware and GNSS antenna installation;
• The data acquisition frequency is limited by the maximum I2C bandwidth instead of the GNSS receiver itself, which is difficult to predict during the design stage;
• Limited information is available for the GNSS hardware, potentially limiting its capabilities in the absence of suitable technical information;
• Unexplained technical difficulties were encountered with streaming correction data from the NTRIP Client Android application, either not working on specific smartphones or periodically terminating transmission altogether for others.

## Funding

4Tel Pty is gratefully acknowledged for sponsoring the Chair in Railway Engineering in the Department of Civil Engineering at the University of Pretoria.

## CRediT authorship contribution statement

**André Broekman:** Conceptualization, Data curation, Formal analysis, Investigation, Methodology, Project administration, Software, Validation, Writing - original draft. **Petrus Johannes Gräbe:** Funding acquisition, Project administration, Resources, Supervision, Validation, Writing – review & editing.

## Declaration of Competing Interest

The authors declare that they have no known competing financial interests or personal relationships that could have appeared to influence the work reported in this paper.
